# Contributions of network structure, chemoarchitecture and diagnostic categories to transitions between cognitive topographies

**DOI:** 10.1038/s41551-024-01242-2

**Published:** 2024-08-05

**Authors:** Andrea I. Luppi, S. Parker Singleton, Justine Y. Hansen, Keith W. Jamison, Danilo Bzdok, Amy Kuceyeski, Richard F. Betzel, Bratislav Misic

**Affiliations:** 1 Montreal Neurological Institute, McGill University, Montreal, Quebec Canada; 2 Department of Computational Biology, Cornell University, Ithaca, NY USA; 3 MILA, Quebec Artificial Intelligence Institute, Montreal, Quebec Canada; 4 Department of Radiology, Weill Cornell Medicine, New York, NY USA; 5 Psychological and Brain Sciences, Indiana University, Bloomington, IN USA

**Keywords:** Computational neuroscience, Cognitive neuroscience, Biomedical engineering

## Abstract

The mechanisms linking the brain’s network structure to cognitively relevant activation patterns remain largely unknown. Here, by leveraging principles of network control, we show how the architecture of the human connectome shapes transitions between 123 experimentally defined cognitive activation maps (cognitive topographies) from the NeuroSynth meta-analytic database. Specifically, we systematically integrated large-scale multimodal neuroimaging data from functional magnetic resonance imaging, diffusion tractography, cortical morphometry and positron emission tomography to simulate how anatomically guided transitions between cognitive states can be reshaped by neurotransmitter engagement or by changes in cortical thickness. Our model incorporates neurotransmitter-receptor density maps (18 receptors and transporters) and maps of cortical thickness pertaining to a wide range of mental health, neurodegenerative, psychiatric and neurodevelopmental diagnostic categories (17,000 patients and 22,000 controls). The results provide a comprehensive look-up table charting how brain network organization and chemoarchitecture interact to manifest different cognitive topographies, and establish a principled foundation for the systematic identification of ways to promote selective transitions between cognitive topographies.

## Abstract (web summary)

An analysis of neuroimaging data across mental health, neurodegenerative, psychiatric and neurodevelopmental diagnostic categories highlights how brain network organization and chemoarchitecture interact to shape cognitive topographies.

## Main

The brain is a complex system of interconnected units that dynamically transitions through diverse activation states supporting cognitive function^1–6^. Large-scale, non-invasive techniques such as functional magnetic resonance imaging (fMRI) provide a way to map activation patterns to cognitive functions^7–12^. Healthy brain function requires the ability to flexibly transition between different patterns of brain activation, to engage the corresponding cognitive functions in response to environmental and task demands. In turn, the neurophysiological dynamics of the human brain are both constrained and supported by the network organization of the structural connectome: the white matter fibres that physically connect brain regions^13–17^. However, the exact mechanisms by which the brain’s network architecture shapes its capacity to transition between cognitively relevant activation patterns remain largely unknown, and an intense focus of inquiry in neuroscience^18–21^.

Network control theory is a computational paradigm that explicitly operationalizes how the architecture and dynamics of a network support transitions between activation states^20,22–24^. Originally developed in the physics and engineering literature^22,25,26^, network control theory conceptualizes the state of a dynamical system at a given time as a linear function of three elements: (i) the previous state, (ii) the structural network linking system units and (iii) input injected into the system to control it.

In the context of the brain, such input can intuitively take the form of task modulation^27–29^ or other perturbations from the environment, but potentially also pharmacological or direct electromagnetic stimulation^30–33^, or endogenous signals from elsewhere in the brain^34^. This approach is widely applicable across the breadth of neuroscience, from *Caenorhabditis* *elegans* and *Drosophila*^20,35^ to rodents and primates^20,23^, and across human development^36–38^, health and disease^27,28,31,39–43^.

In humans, network control can be used to study the transition between brain states. Such state-to-state transitions can be formalized as a dynamical process that unfolds over the connectome’s network architecture, reconstructed from diffusion-weighted imaging (DWI). Of particular relevance is the quantification of control energy. Control energy refers to the magnitude of input that needs to be provided to the system to drive its trajectory from an initial state to a desired target state^22,23^. In the context of transitions between brain states, the cost of transitions may correspond to the magnitude of exogenous stimulation (for example, transcranial magnetic stimulation, deep brain stimulation, intracranial stimulation^30,31,44,45^ or the dose of a pharmacological intervention)^27,33,46^, but also to endogenous effort, as reflected by cognitive demand^34,47^.

When seeking to operationalize this framework, conventional studies on control energy in the human brain have consistently adopted one of two strategies for defining brain states. One strategy is to define brain states as co-activations of cognitively relevant brain circuits, operationalized as the canonical intrinsic connectivity networks of the brain^28,29,43,48,49^. The downside of this approach is that intrinsic networks identified from fMRI are limited in number (usually only 7–8)^3,50–53^, providing a correspondingly limited repertoire compared with the space of possible functional activation patterns. The second strategy typically involves defining brain states as random activation patterns^20,43^, whose number is then virtually limitless, but at the expense of being cognitively ambiguous.

Here we overcome these challenges by investigating how network architecture supports transitions between cognitive topographies. We define cognitive topographies (that is, cognitively relevant brain states) as meta-analytic patterns of cortical activation pertaining to over 100 cognitive terms, obtained by aggregating over 14,000 fMRI studies from the NeuroSynth atlas^8^. This approach represents a large-scale generalization of recent work that defined cognitively relevant brain states in terms of task-based fMRI contrast maps^27,54^.

In addition to generalizing the set of possible start and target states under consideration, we also provide two key extensions to the scope of the control inputs under investigation. First, we consider the potential role of changes in the capacity of brain regions to act as sources of endogenous control signals, associated with a variety of diagnostic categories.

We operationalize this using cortical thickness changes for 11 neurological, psychiatric and neurodevelopmental diagnostic categories from the ENIGMA consortium, summarizing contrasts between 17,000 patients and 22,000 controls^55–58^. Second, inspired by recent work^33,59^, we extend our computational framework to approximate the effects of engaging different neurotransmitter systems. Since many pharmacological agents exert their effects on the brain by engaging neurotransmitter receptors and transporters, this approach enables us to investigate the potential role of pharmacological perturbations on cognitive transitions. Specifically, we define regionally heterogeneous inputs as the regional expression of 18 neurotransmitter receptors and transporters, quantified from in vivo positron emission tomography (PET) scans in >1,200 participants^60^. Overall, we combine multiple databases from different neuroimaging modalities (fMRI, diffusion MRI (dMRI) tractography, cortical morphometry and PET) to investigate how the brain’s network architecture shapes its capacity to transition between a large number of experimentally defined cognitive topographies, and how this capacity can be reshaped by engaging neurotransmitter systems or by changes in cortical thickness.

## Results

**Fig. 1:**
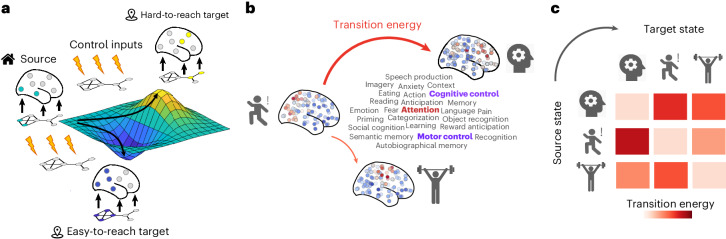
Network control with cognitive topographies. **a**, Functional brain activity (coloured nodes are active; grey nodes are inactive) evolves through time over a fixed network structure (shown below the brains). From a given starting configuration of activity (green), some alternative configurations are relatively easy to reach in the space of possible configurations (valley, in blue), whereas others are relatively difficult to achieve (peak, in yellow). To reach a desired target configuration of activity, input energy (represented by the lightning bolt icons) can be injected locally into the system, and this energy will spread to the rest of the system based on its network organization. **b**, We define states as 123 meta-analytic activation maps from the NeuroSynth database. We then use network control theory to quantify the cost of transitioning between these cognitive topographies. **c**, Systematic quantification of transition cost between each pair of cognitive topographies results in a look-up table mapping the energy required for each transition.

Network control allows us to ask how the brain network structure supports transitions between cognitively relevant brain states^23,34^ (Fig. 1a). We define the network whose activity is to be controlled as the human structural connectome (obtained as a consensus of *N* = 100 Human Connectome Project (HCP)^61^ participants’ connectomes reconstructed from dMRI tractography; Methods). We define the cognitive topographies as meta-analytic brain activation patterns from the NeuroSynth atlas (Methods). With definitions of the network and its state in hand, we consider the problem of network controllability: how can the system be driven to specific target states by internal or external control inputs (Fig. 1b)? Beginning with uniform inputs applied to all regions, we are interested in the relative energetic cost of transitions between different cognitive topographies (Fig. 1c).

### Transitions between cognitive topographies

We first evaluate the control energy required to transition between each pair of cognitive topographies (‘brain states’) from NeuroSynth^8^. The NeuroSynth meta-analytic engine provides meta-analytic functional activation maps associated with 123 cognitive and behavioural terms from the Cognitive Atlas^62^, ranging from general terms (‘attention’ and ‘emotion’) to specific cognitive processes (‘motor control’ and ‘autobiographical memory’), behavioural states (‘eating’ and ‘sleep’) and emotional states (‘fear’ and ‘anxiety’). Each map is a vector encoding the statistical strength of differential activation for fMRI experiments that include a cognitive term versus studies that do not include that term, at each spatial location, based on the published literature. Although NeuroSynth uses activation maps as the inputs for its term-based meta-analysis, its outputs are not activation maps per se, but rather they reflect statistical association tests.

**Fig. 2:**
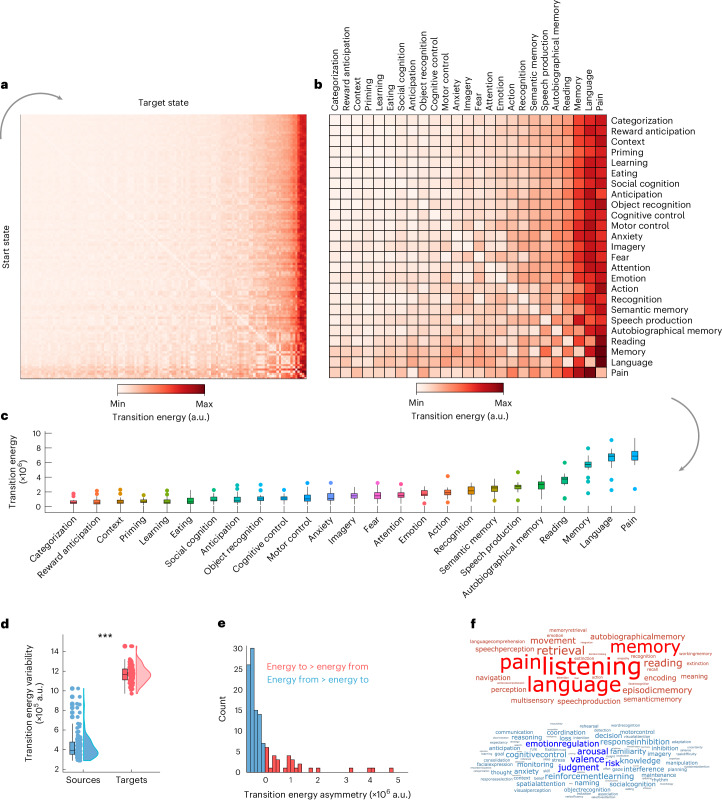
Quantifying transitions between cognitive topographies. **a**, Transition cost (energy) between each pair of 123 cognitive topographies (‘states’) from NeuroSynth. Rows indicate source states; columns indicate target states. **b**, Same as **a**, but showing only a subset of 25 out of 123 NeuroSynth states for visualization purposes. Matrices are sorted by increasing cost across both rows and columns. **c**, Distributions of the cost to transition to each cognitive topography from every other cognitive topography. Box plot: centre line, median; box limits, upper and lower quartiles; whiskers, 1.5× interquartile range; points, outliers. **d**, Variability (s.d.) of transition energy is greater along the column dimension (target states) than along the row dimension (source states). This effect is also observed for the subset of 25 terms (Supplementary Fig. 1; ***P < 0.001). Box plot: centre line, median; box limits, upper and lower quartiles; whiskers, 1.5× interquartile range; points, outliers. **e**, Histogram of the difference in transition cost between reaching each state (averaging across all possible source states) and leaving each state (averaging across all possible target states). Positive values indicate greater cost to reach a state than to leave it, whereas negative values indicate the reverse. **f**, Word clouds show the NeuroSynth terms that are more difficult to reach than to leave, on average (red), or more difficult to leave than to reach, on average (blue). Word size reflects ranking. Source data are provided as a Source Data file. Source data

**Extended Data Fig. 1:**
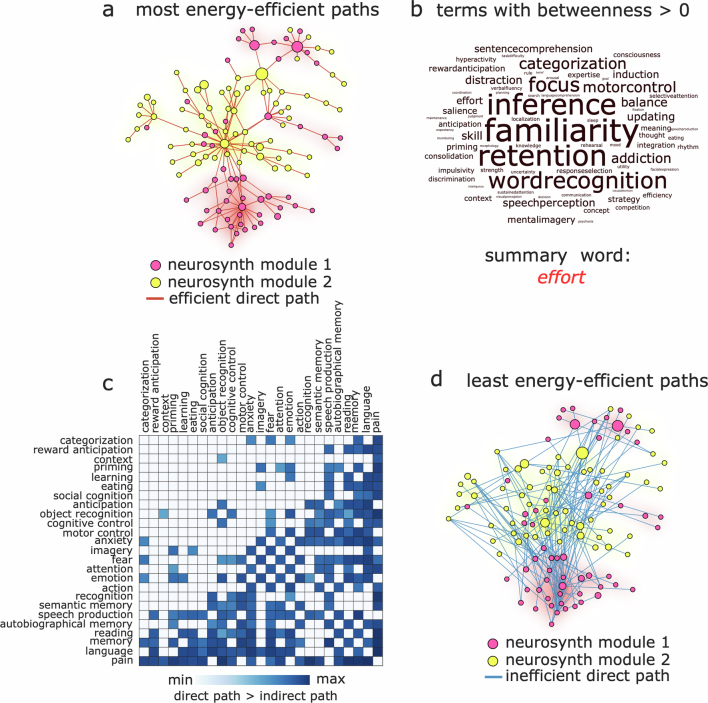
Efficient and inefficient paths between cognitive topographies. (**a**) Representation of the control energy matrix as a weighted network, showing the transitions (edges) between cognitive topographies (nodes) that require the least energy (for display purposes, only 10% of connections are shown). Nodes are colored according to their membership of the two communities into which cognitive topographies cluster. Cognitive topographies that act as intermediaries for the most efficient transition between two other cognitive topographies correspond to nodes that have non-zero betweenness centrality. Node size reflects the betweenness centrality of each node (taking into account all paths). (**b**) The NeuroSynth terms corresponding to cognitive topographies with non-zero betweenness centrality in the network representation in (a); size reflects the betweenness centrality of the corresponding nodes. To identify the term that most summarises all others, we represent each term as a highdimensional vector in semantic space using word2vec [201], and we measure their similarity using cosine similarity between these vector representations. We find that ‘effort’ has the highest mean cosine similarity with the vector representations of all other high-betweenness terms. (**c**) Matrix of the transition cost between each pair of cognitive topographies in the reduced set, showing the difference in control energy between the direct and least-expensive paths; the value of each non-empty cell indicates the energy premium incurred by taking the direct path between two cognitive topographies (log-transformed to better show the distribution). (**d**) The most costly direct paths between cognitive topographies (only 10% shown, for display purposes). Node size, colour, and position are the same as in (a). Source data are provided as a Source Data file. Source data

Applying control inputs uniformly to all brain regions, we compute the optimal energy cost for each of the 15,129 possible transitions between cognitive topographies. We find that optimal control energy can vary by nearly tenfold across different transitions (Fig. 2a–c). As a result of these differences, for several combinations of source and target cognitive topographies (36%), a direct transition is not the most energy-efficient. Rather, the control energy required to transition between them can be reduced if an intermediate transition is made to some other state (Extended Data Fig. 1). Of note, the cortical topographies associated with these intermediate states converge on the default mode network (DMN), especially its posterior hubs of left posterior cingulate/precuneus and bilateral angular gyrus (Supplementary Fig. 2).

**Extended Data Fig. 2:**
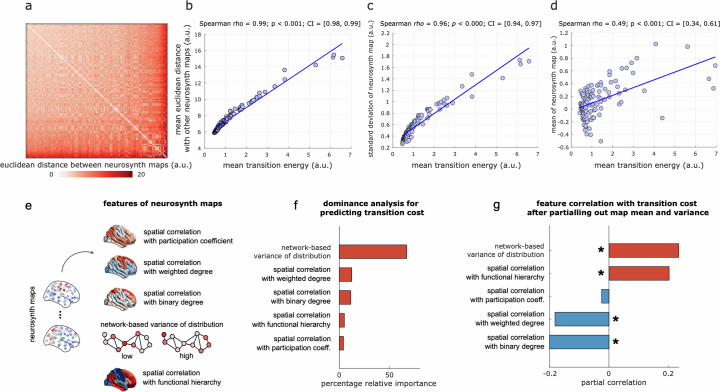
Predictors of transition energy. (**a**) Euclidean distance between the vectors corresponding to each NeuroSynth map. (**b**) Transition energy to a given cognitive topography (averaged over all starting states) correlates with the mean Euclidean distance between its corresponding NeuroSynth map and all others (p < 0.001 from Spearman correlation, two-sided). (**c**) Transition energy to a given cognitive topography (averaged over all starting states) correlates with the standard deviation of its NeuroSynth map (p < 0.001 from Spearman correlation, two-sided). (**d**) Transition energy to a given cognitive topography (averaged over all starting states) correlates with the mean of its NeuroSynth map (p < 0.001 from Spearman correlation, two-sided). (**e**) Mean and variance of a NeuroSynth map are coarse descriptions that do not account for neuroanatomy. As neuroanatomically-grounded predictors of transition energy, we consider the following features of each NeuroSynth map: (i) the map’s spatial alignment with the regional distribution of participation coefficients, treating the structural connectome as a network; (ii) the map’s spatial alignment with the regional distribution of weighted node degree, treating the structural connectome as a network; (iii) the map’s spatial alignment with the regional distribution of binary node degree, treating the structural connectome as a network; (iv) a recently developed measure of variance for distributions over a network [90, 153]: unlike the usual measure of variance, which assumes independent data-points and is agnostic to their spatial location, this measure takes into account the relationships between observations. Specifically, variance of a distribution over a network is low, if high values occur at nodes that are easy to reach using a diffusion process along network paths of all length. Conversely, if the majority of high values occurs at nodes that are difficult to reach using diffusion, then the distribution will have high network-based variance. This is especially relevant because network control theory operationalises control inputs as spreading by diffusing over the network. (v) The map’s spatial alignment with the principal gradient of functional connectivity (unimodal-transmodal hierarchy) [152]. (**f**) Bar plot shows the relative dominance of each predictor as obtained from dominance analysis [155]. Dominance analysis distributes the fit of the model across predictors such that the contribution of each predictor can be assessed and compared to other predictors, reflecting the proportion of the variance jointly explained by all predictors, that can be attributed to each predictor. (**g**) Bar plot shows the partial correlation between each predictor and the average cost to transition to a given brain state, after controlling for the effects of brain state mean and variance. *, p < 0.05 from partial correlation (two-sided; see Source Data for full statistical reporting). Source data are provided as a Source Data file. Source data

The transition energy between each pair of states correlates with the Euclidean distance between their NeuroSynth vector representations (Spearman’s *r* = 0.99, *P* < 0.001): the more distant two patterns are, the more energy will be required, and consequently the average energy to reach each target state correlates with the mean (Spearman’s *r* = 0.49, *P* < 0.001) and standard deviation (s.d.) (Spearman’s *r* = 0.96, *P* < 0.001) of the corresponding NeuroSynth map (Extended Data Fig. 2a–d).

However, mean and variance of the NeuroSynth activation patterns are coarse descriptions of each pattern. This is because they disregard all information about the neuroanatomical distribution of activations. In addition, Euclidean distance alone cannot fully account for the observed results: Euclidean distance is symmetric, whereas we observe that transition energy is asymmetric. Specifically, we observe that target states (columns of the matrix) exhibit greater variability than start states (rows), suggesting that the destination of a transition—the desired target topography—may play a more prominent role than the current state in determining the ease or difficulty of the transition (Fig. 2a–c). After partialling out the effect of each NeuroSynth map’s mean and s.d., we find that its transition cost is related to both topological features of the structural connectome (especially whether high-valued nodes are easy or difficult to reach using a diffusion process), and the map’s spatial alignment with the unimodal–transmodal cortical hierarchy (Extended Data Fig. 2e–g).

We confirm the observation of transition asymmetry by showing that the variability (s.d.) of the transition energy matrix is higher across target states (mean = 1.17 × 10^6^, s.d. = 8.74 × 10^4^) than across start states (mean = 4.35 × 10^5^, s.d. = 1.35 × 10^5^; *t*(244) = 50.52, *P* < 0.001, Cohen’s *d* = 6.42) (Fig. 2d). To investigate this asymmetry further, we also compute a measure of transition asymmetry between each pair of brain states *i* and *j*, as the difference in control energy required to move from *i* to *j*, versus moving from *j* to *i*. Averaging across start states provides, for each target state, a measure of whether that brain state is overall easier to reach than leave (negative values) or harder to reach than leave (positive values) from other states. As expected, this measure is positively correlated with the overall transition cost to reach a given state (Spearman’s *r* = 0.73, *P* < 0.001). We find that the majority of cognitive topographies are slightly easier to reach than to leave, but this is counterbalanced by a small number of cognitive topographies that are substantially harder to reach than to leave. In particular, hard-to-reach cognitive topographies include those pertaining to language-related cognitive operations (for example, ‘language’, ‘reading’ and ‘speech production’) and those pertaining to memory (for example, ‘memory’, ‘autobiographical memory’ and ‘semantic memory’) (Fig. 2e,f). We confirm this observation quantitatively via empirical permutation tests (1,000 permutations): we consider the six data-driven cognitive domains identified by Beam and colleagues^11^: memory, cognition, inference, emotion, vision and language. Among the terms in our list of 123 that have been assigned to one of these six domains, we find that terms pertaining to ‘memory’ (*P* = 0.007) and ‘language’ (*P* = 0.045) exhibit a higher median value of asymmetry than would be expected by chance (‘emotion’, *P* = 0.640; ‘inference’, *P* = 0.696; ‘cognition’, *P* = 0.542; ‘vision’, *P* = 0.730).

It is possible that the presence of ‘nested’ terms among our 123 NeuroSynth terms may skew the distribution of transition energies, by making it seem as though the average energy required to transition to cognitive topographies pertaining to such terms is lower. However, we show that when these ‘nested’ terms are removed, leaving only the most general one for each family of terms, the global pattern of transitions is still preserved: the ranking of cognitive topographies in terms of average transition energy remains unchanged (Spearman’s r = 1.0, *P* < 0.001) when nested terms are included as sources or excluded (Supplementary Fig. 3). This indicates that the presence of overlapping and nested terms is not changing the distribution of which cognitive topographies are more or less difficult to reach.

The NeuroSynth terms included all pertain to some specific cognitive state or operation. However, it is also of interest whether similar transition costs would be observed when starting from a state of baseline. We find that this is indeed the case. We operationalize such a baseline using a map of regional cerebral blood flow^63^ and compute the transition energy from this baseline to every cognitive topography from NeuroSynth. Our results show that the average transition energy is similar, whether one is starting from other cognitive topographies or from this ‘baseline’ state (Supplementary Fig. 4). In other words, states that are difficult to reach when switching from other cognitive states are also difficult to reach when starting from a baseline that corresponds to no specific cognitive state. Altogether, we find that the energetic ease or difficulty of transitioning between two given cognitive topographies appears to be primarily driven by the identity of the target state.

### Connectome wiring supports efficient cognitive transitions

Having considered how transition energy varies as a function of different origin and target cognitive topographies, we now turn our attention to the network itself. We assess how much the observed effects are due to topology and geometric embedding. To address this question, we implement two classes of null models^64^.

We first consider a null model that preserves weight distribution and degree sequence^65^, and a ‘geometry preserving’ rewired null, preserving degree sequence and weight distribution, but also the approximate wiring cost (length of connections)^66^. For each specific null model, we re-estimate the control energy 500 times and compare the resulting distribution of all-to-all mean transition energy against the distribution obtained using the empirical structural connectome of the human brain.

**Fig. 3:**
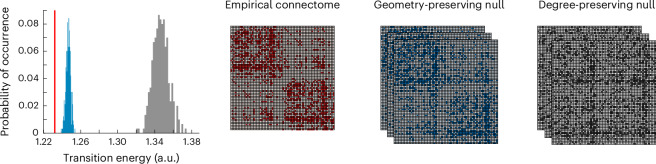
Role of network topology in supporting transitions between cognitive topographies. Degree-preserving randomized null models (grey) and null models that preserve the exact degree sequence and the approximate length distribution (blue) are significantly less favourable than the empirical human connectome (red) to support transitions between cognitive topographies. Note that all networks are weighted and have the exact same distribution of weights, but they are shown here as binary to highlight the respective similarities and differences in topology. Source data are provided as a Source Data file. Source data

We find that the human brain outperforms both null models: transitions are significantly less energy expensive on the human connectome than on either null (all *P* < 0.001; Fig. 3), suggesting that the unique wiring architecture of the human connectome supports efficient transitions between cognitively relevant topographies. We also find a significant difference between the nulls: rewired networks are more energy-efficient when they preserve both the degree sequence and the geometric properties of the human connectome, than when only the degree sequence is preserved. In other words, the geometric embedding of the human connectome accounts for a substantial portion of its energy efficiency—but not all of it. We also show that these observations, obtained from a consensus connectome, remain true at the single-participant level: participant-level matrices of transition energy correlate with the group-level matrix, and individual connectomes outperform corresponding degree-preserving and degree- and cost-preserving nulls (Supplementary Fig. 5 and Supplementary Table 1).

### Effect of diagnostic categories on transitions

Up to this point, we investigated both the role of the transition sources and targets, and the role of the underlying network. We now turn to the remaining element of the network control theory as a framework for dynamics on neuronal networks: the control inputs. Although uniform control represents the simplest case, we can also consider the case where intrinsic control input is heterogeneous across regions, according to some property of each region^33^. Here we consider heterogeneity in terms of regional cortical thickness, with the rationale that all else being equal, a region of thicker grey matter may be expected to send more endogenous input signals to the rest of the system through its connections. This intuition is based on the simplifying assumption that the amount of endogenous input energy that a region is capable of contributing to the network should be a function of its total abundance of excitatory neurons (since most inter-regional projections are known to originate from excitatory neurons)—and that this, in turn, would be related to cortical thickness (since most neurons are excitatory). This approach is conceptually related to the approach recently used by Singleton and colleagues^33,59^, who applied non-uniform control inputs according to the normalized density of the serotonin 2A receptor expressed in each region (as quantified by PET). We show that the overall transition cost between our 123 cognitive topographies is similar when using uniform control inputs, or heterogeneous control inputs provided by the map of regional cortical thickness of the healthy human brain (rescaled to have unit mean to be comparable with the uniform control model) (Supplementary Fig. 6).

**Fig. 4:**
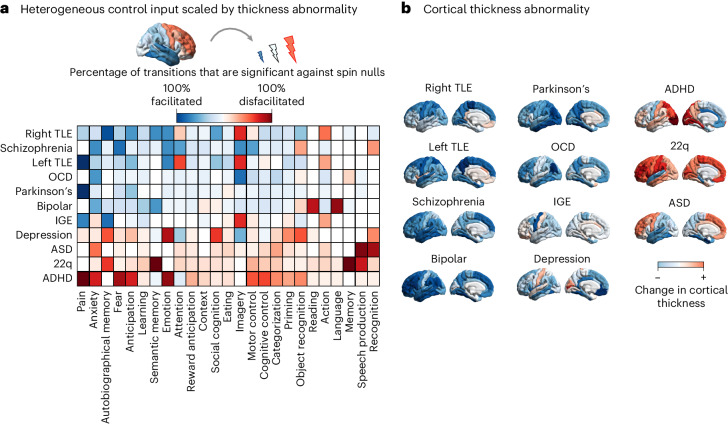
Simulating the effect of changes in cortical thickness associated with diagnostic categories. **a**, The effect of cortical thickness changes is modelled by varying the control input provided by each region, in proportion to the extent of its cortical thickness alteration from healthy controls (Cohen’s *d*): atrophied regions exert less input, and regions of increased thickness exert greater input. Heatmap shows how each diagnostic category reshapes the transition energy required to reach a given cognitive topography, presented as the percentage of transitions (out of all possible initial cognitive topographies) that are significantly facilitated (blue colour scale) or significantly disfacilitated (red colour scale). Significance is assessed against a null distribution of randomly rotated cortical thickness alteration maps with preserved mean, variance and spatial autocorrelation, such that the only differences with the original map are the neuroanatomical locations of increases and decreases. **b**, The changes in cortical thickness associated with each diagnostic category are shown on the cortical surface. ADHD, attention deficit hyperactivity disorder; ASD, autistic spectrum disorder; OCD, obsessive-compulsive disorder; IGE, idiopathic generalized epilepsy; right TLE, right temporal lobe epilepsy; left TLE, left temporal lobe epilepsy. Source data are provided as a Source Data file. Source data

Next, we consider how changes in cortical thickness associated with different diagnostic categories may impact the connectome’s ability to facilitate transitions between cognitive topographies. As above, our intuition is that all else being equal, an atrophied region should be less capable of engaging with the rest of the brain and providing endogenous control signals to it—and vice versa for an enlarged region. We consider different patterns of changes in cortical thickness associated with 11 neurological, neurodevelopmental and neuropsychiatric diagnostic categories as quantified by the ENIGMA consortium and recent related publications^55–58^. For each of the 11 diagnostic categories, we modulate the regional control input according to the regional pattern of increases or decreases in cortical thickness associated with that condition (Fig. 4). In other words, when a decrease of thickness is observed, we model it as a decrease in control input, and vice versa when an increase is observed.

The overall difference in the cost of all pairwise transitions, compared with baseline (uniform control), is shown in Supplementary Fig. 7. We observe that most diagnostic categories from the ENIGMA database incur greater transition costs than baseline (where baseline corresponds to uniform control energy). This may be attributed to the fact that for most diagnostic categories (except autism, ADHD and 22q11.2 deletion syndrome), the majority of regions exhibit reduced cortical thickness, rather than increases (Fig. 4b). Since in our model this corresponds to reduced control input being provided to the system, the overall control input is diminished, and thus the transition cost is higher.

However, it is important to disentangle the role of overall changes in cortical thickness, versus their specific neuroanatomical distribution. To this end, and to remove the potential confound of the mean and variance of each distribution, we compare the transition energy associated with each pattern of cortical thickness, against a null distribution of randomly rotated versions of the same pattern, preserving the original brain map’s mean, variance and spatial autocorrelation, but randomizing the neuroanatomical locations^67^. We define statistically significant facilitation as occurring when a transition requires less energy with the empirical map of cortical thickness changes than with a null population of randomized maps with preserved spatial autocorrelation, mean and variance. Conversely, we define statistically significant disfacilitation as occurring when a transition requires more energy with the empirical map of cortical thickness changes than with a null population of randomized maps with preserved spatial autocorrelation, mean and variance. Therefore, it would be possible for a set of cortical thickness changes to increase control energy when compared against uniform control, but reduce it when compared against a distribution of mean-, variance- and autocorrelation-preserving null maps. Such a result would indicate that the impact of the cortical thickness changes is less severe than would be expected based on random occurrence of the same changes in the brain.

This approach allows us to determine the relevance of increases and decreases in cortical thickness occurring at specific regions for reshaping transition costs. Importantly, this approach does not imply that changes in cortical thickness are the cause of a given condition. Rather, given that cortical thickness changes have occurred (whether as cause or consequence), we seek to evaluate what associations they have and what role they may play in reshaping the energetic cost of transitioning between cognitive topographies. Our results show that for some diagnostic categories, such as schizophrenia, bipolar disorder or the temporal lobe epilepsies, the neuroanatomical distribution of cortical changes is such that transition costs are on average lower than would be expected by only considering equivalent but randomly distributed changes in cortical thickness. By contrast, other conditions such as autism, ADHD, depression and 22q syndrome incur transition costs that exceed what would be expected based on randomly distributed cortical thickness increases and decreases (Fig. 4). We also show that the spatial correlations between ENIGMA maps and cognitive topographies do not trivially predict how ENIGMA maps reshape the mean transition energy (*r* = −0.13, *P* = 0.091; Supplementary Fig. 8).

### Effect of engaging neurotransmitter systems

Finally, inspired by recent work that applied regionally heterogeneous control inputs according to PET-derived regional expression of serotonin receptors^33,59^, we extend our computational framework to approximate the effects of engaging different neurotransmitter systems.

**Fig. 5:**
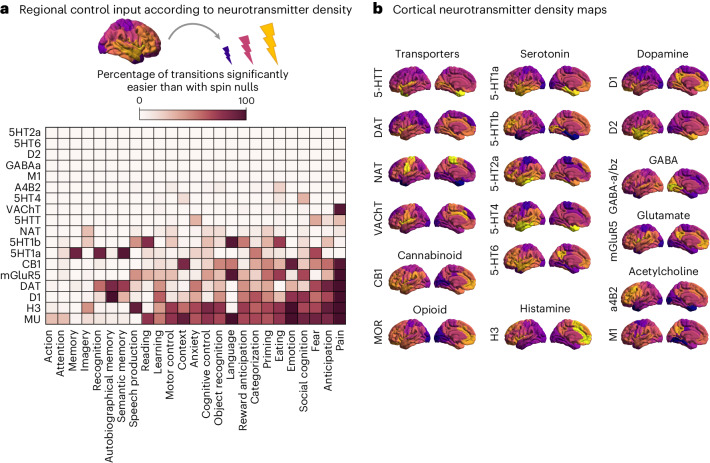
Modelling how neurotransmitter systems can reshape transitions. **a**, The effect of engaging each neurotransmitter receptor and transporter is modelled by changing the control input provided by each region in proportion to its density of receptor/transporter expression, measured by in vivo PET. For each receptor/transporter, the heatmap shows the percentage of transitions to each cognitive state (out of the possible start states) that require significantly less energy when using the empirical PET map, than when using null maps with preserved spatial autocorrelation and distribution of values, but occurring at different neuroanatomical locations. **b**, The empirical spatial distribution of each receptor and transporter is shown on the cortical surface. Source data are provided as a Source Data file. Source data

Therefore, we modulate control inputs in proportion to the regional expression of different neurotransmitter receptors and transporters, quantified from in vivo PET. We consider a total of 18 recently assembled PET maps^60^. This allows us to evaluate how engaging each receptor—based on its regional distribution—favours transitions towards different cognitive topographies. As above, we account for the overall distribution of values in each receptor map by comparing it with randomly rotated null maps having the same distribution of values and spatial autocorrelation, but different association with neuroanatomy^67^. Thus, we define statistically significant transitions as those that require less energy using empirically estimated maps of neurotransmitter receptors than randomized maps with preserved spatial autocorrelation, mean and variance. Our results show that some cognitive topographies are more susceptible than others to facilitation via receptor-informed stimulation, whereas others exhibit little benefit (Fig. 5).

In addition, we find differences between receptors in terms of their propensity to facilitate transitions, over and above the mere effect of increased input (that is, performing better than randomly rotated counterparts). Specifically, the dopamine transporter and D_1_ receptor, the mu-opioid receptor and the histamine H_3_ receptor maps performed best (Fig. 5). Our computational framework identifies these receptors and transporters as those whose neuroanatomical distribution is mostly suited to facilitate transitions towards a variety of cognitive topographies.

### Sensitivity and robustness

**Extended Data Fig. 3:**
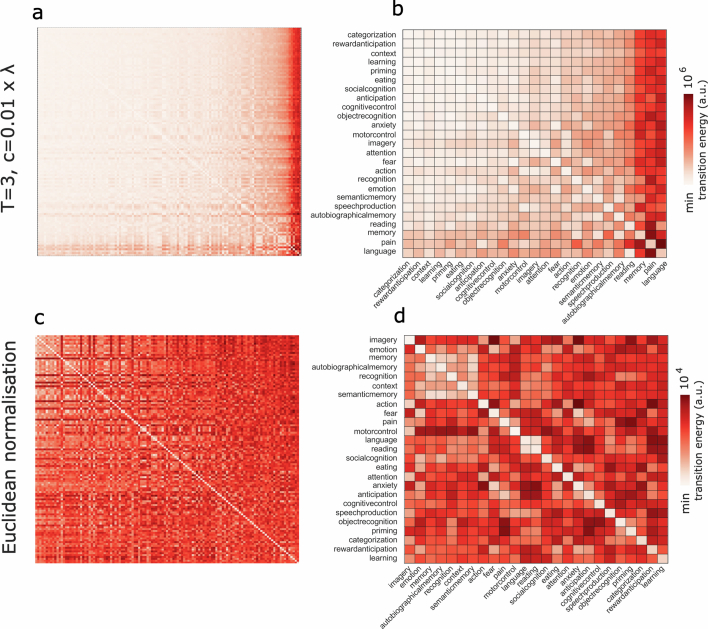
Transition energies for alternative operationalisations of network control theory. (**a**, **b**) Transition energy between each pair of 123 cognitive topographies from NeuroSynth (a) and the reduced set of 25 NeuroSynth terms (b), for network control with time horizon T = 3 and network normalisation factor c = 0.01 × |λ(A)max|. Rows indicate source states, columns indicate target states. (**c**, **d**) Transition energy between each pair of 123 cognitive topographies from NeuroSynth (c) and the reduced set of 25 NeuroSynth terms (d), for network control with all NeuroSynth maps normalised to unit Euclidean norm. Source data are provided as a Source Data file. Source data

**Extended Data Fig. 4:**
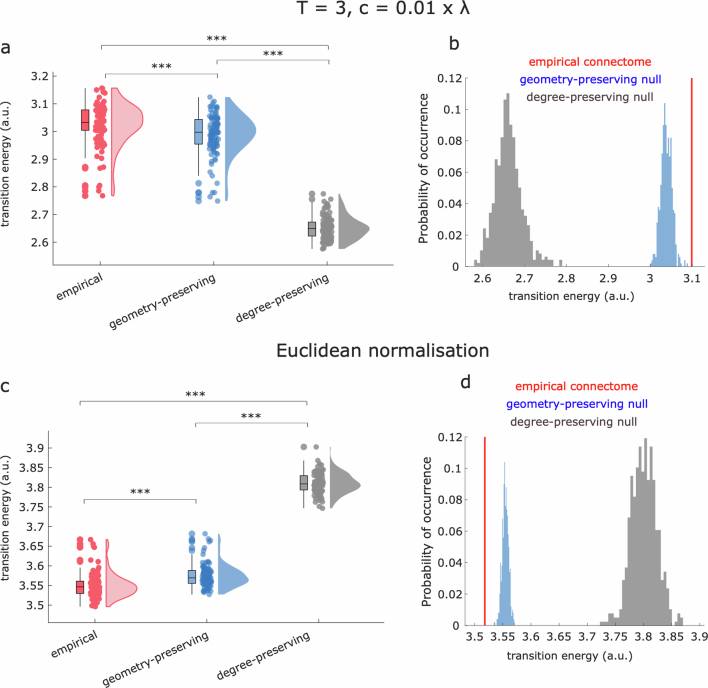
Role of network topology in supporting transitions, for alternative operationalisations of network control theory. (**a**, **b**) participant-wise (a) and group-level (b) average transition energy for the empirical human connectome (red) and for degree-preserving (grey) and degree- and cost-preserving null models (blue), for network control with time horizon T = 3 and network normalisation factor c=0.01 × |λ(A)max|. n = 100 participants and corresponding null networks. Box-plots: center line, median; box limits, upper and lower quartiles; whiskers, 1.5× interquartile range; ***, p < 0.001 from paired-samples t-tests (two-sided). (**c**, **d**) participant-wise (c) and group-level (d) average transition energy for the empirical human connectome (red) and for degree-preserving (grey) and degree- and cost-preserving null models (blue), for network control with all NeuroSynth maps normalised to unit Euclidean norm. Source data are provided as a Source Data file. Source data

**Extended Data Fig. 5:**
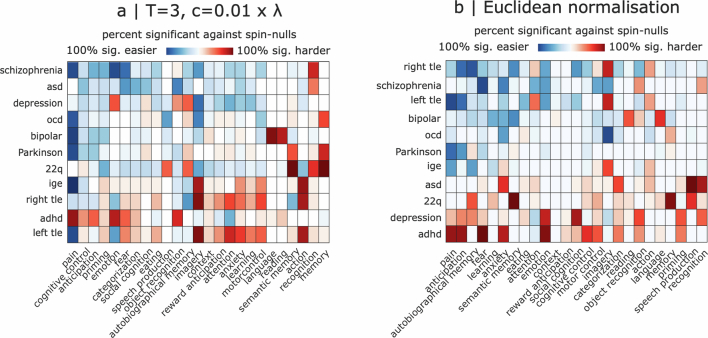
Effects of cortical thickness changes associated with each diagnostic category, for alternative operationalisations of network control theory. Heatmap shows how each diagnostic category reshapes the transition energy required to reach a given cognitive topography, presented as the percentage of transitions (out of all possible initial cognitive topographies) that are significantly facilitated (blue colour scale) or significantly dis-facilitated (red colour scale). Significance is assessed against a null distribution of randomly rotated cortical thickness alteration maps with preserved mean, variance, and spatial autocorrelation, such that the only differences with the original map are the neuroanatomical locations of increases and decreases. (**a**) For network control with time horizon T = 3 and network normalisation factor c = 0.01 × |λ(A)max | ; (**b**) for network control with all NeuroSynth maps normalised to unit Euclidean norm. adhd = attention deficit/hyperactivity disorder; asd = autistic spectrum disorder; ocd = obsessive-compulsive disorder; ige = idiopathic generalised epilepsy; right tle = right temporal lobe epilepsy; left tle = left temporal lobe epilepsy. Source data are provided as a Source Data file. Source data

**Extended Data Fig. 6:**
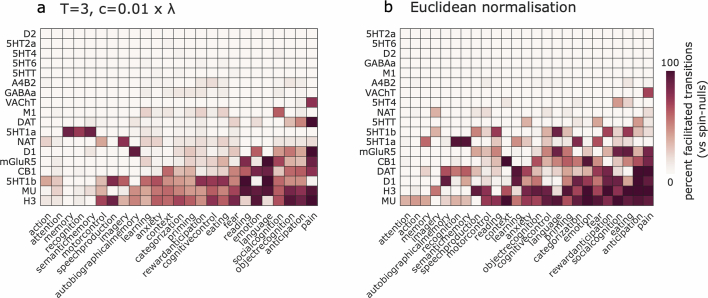
How neurotransmitter systems can reshape the energy landscape of the human brain, for alternative operationalisations of network control theory. Heatmaps show how each receptor/transporter reshapes the average cost of reaching a given cognitive brain state from all other states, as a percentage of transitions to each state that are facilitated, when compared against a null distribution of randomly rotated maps with preserved spatial autocorrelation and the same receptor/transporter density levels, but occurring at different neuroanatomical locations. (**a**) For network control with time horizon time horizon T = 3 and network normalisation factor c = 0.01 × |λ(A)max|; (**b**) for network control with all NeuroSynth maps normalised to unit Euclidean norm. Source data are provided as a Source Data file. Source data

**Extended Data Fig. 7:**
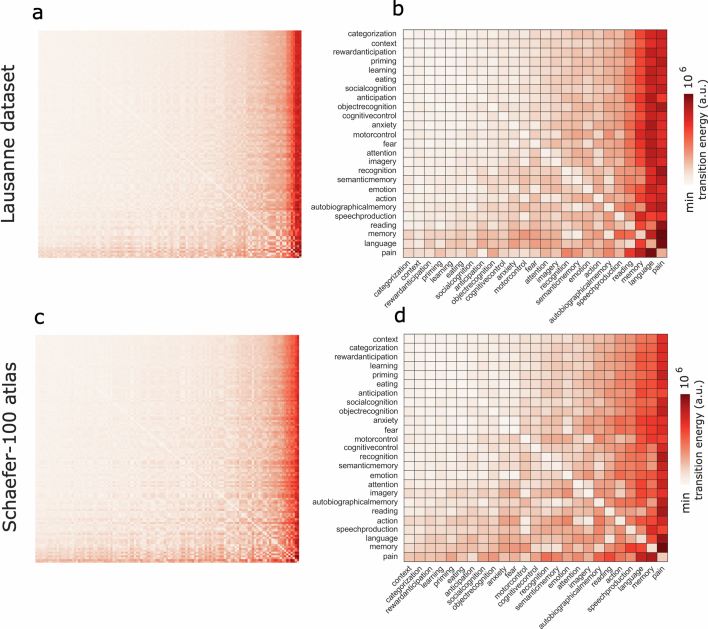
Transition energies for alternative connectome dataset and alternative cortical parcellation. (**a**, **b**) Transition energy between each pair of 123 cognitive topographies from NeuroSynth (a) and the reduced set of 25 NeuroSynth terms (b), for the Lausanne DSI dataset. Rows indicate source states, columns indicate target states. (**c**, **d**) Transition energy between each pair of 123 cognitive topographies from NeuroSynth (c) and the reduced set of 25 NeuroSynth terms (d), for Human Connectome Project data parcellated using the Schaefer-100 cortical atlas. Source data are provided as a Source Data file. Source data

We repeated our analyses using different parameter settings for network control theory, adopting different reconstructions of the human connectome (with a different parcellation, in a separate DWI dataset, using functional instead of structural networks and in a tenfold larger sample), and using two different ways of defining cognitive topographies: based on the expert-curated BrainMap database^7,68^ and based on in-scanner task contrasts for each individual. The Supplementary Information shows results with different implementations of the controllability framework^48^: we consider the time horizon *T* for control, adjacency matrix normalization factor *c* and the effect of normalizing each map to unit Euclidean norm (Extended Data Figs. 3–6 and Supplementary Tables 2 and 3). We also show that our results can be replicated using a different reconstruction of the empirical human connectome, obtained from diffusion spectrum imaging (DSI) (Lausanne consensus dataset)^69^, and using a functional rather than anatomical parcellation of the human cerebral cortex to define network nodes (Schaefer-100)^70^ (Extended Data Fig. 7).

**Extended Data Fig. 8:**
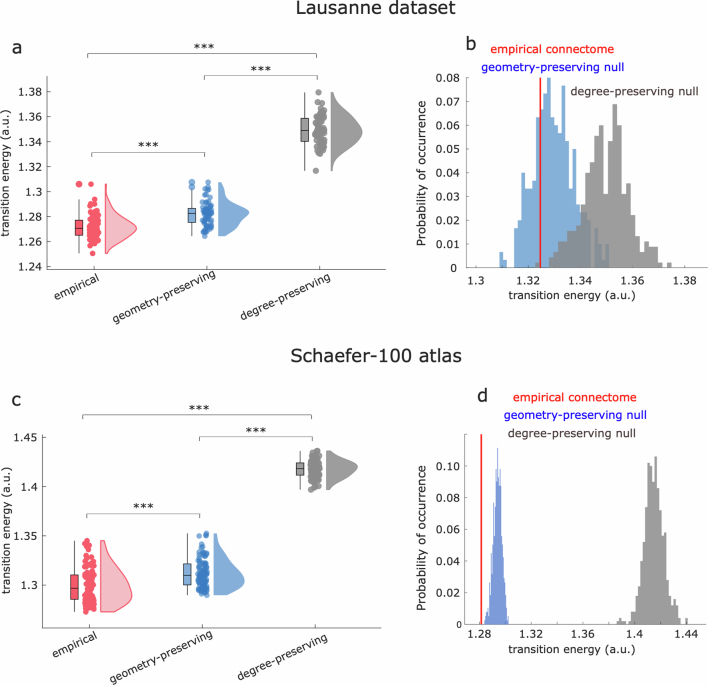
Role of network topology in supporting transitions, for alternative connectome dataset and alternative cortical parcellation. (**a**, **b**) participant-wise (a) and group-level (b) average transition energy for the empirical human connectome (red) and for degree-preserving (grey) and degree- and cost-preserving null models (blue), for the Lausanne DSI dataset (n = 70 participants). Box-plots: center line, median; box limits, upper and lower quartiles; whiskers, 1.5× interquartile range; ***, p < 0.001 from paired-samples t-tests (two-sided). (**c**, **d**) participant-wise (c) and group-level (d) average transition energy for the empirical human connectome (red) and for degree-preserving (grey) and degree- and cost-preserving null models (blue), for Human Connectome Project data parcellated using the Schaefer-100 cortical atlas. Source data are provided as a Source Data file. Source data

Using the Lausanne consensus connectome, we see that the group matrices of transition energy obtained using the HCP and Lausanne consensus connectomes are positively correlated (Spearman’s *r* = 0.99, *P* < 0.001). We also confirm that transitions between cognitive topographies are more energy-efficient on the human connectome than on degree-preserving nulls (*P* = 0.003). Although we do not find a significant difference between the empirical human connectome and the distribution of null networks preserving both degree and wiring cost (*P* = 0.266), the superior energy efficiency of the human connectome is confirmed at the single-participant level (Extended Data Fig. 8a,b and Supplementary Table 4). Both group-level and participant-level results are also replicated with network nodes defined by the Schaefer-100 atlas (Extended Data Fig. 8c,d and Supplementary Table 5).

The Lausanne dataset recapitulates the results about simulating pharmacological intervention (Supplementary Fig. 9a). For Schaefer-parcellated data, we also observe a prominent role of D_1_ receptor and dopamine transporters, as before, but also acetylcholine and noradrenaline transporters (Supplementary Fig. 9b). Lausanne dataset results are also consistent with HCP results in terms of the impact of cortical thickness increase and decrease patterns (note that this analysis could not be repeated with the Schaefer-100 atlas, since ENIGMA data are available only in a single parcellation) (Supplementary Fig. 10).

**Extended Data Fig. 9:**
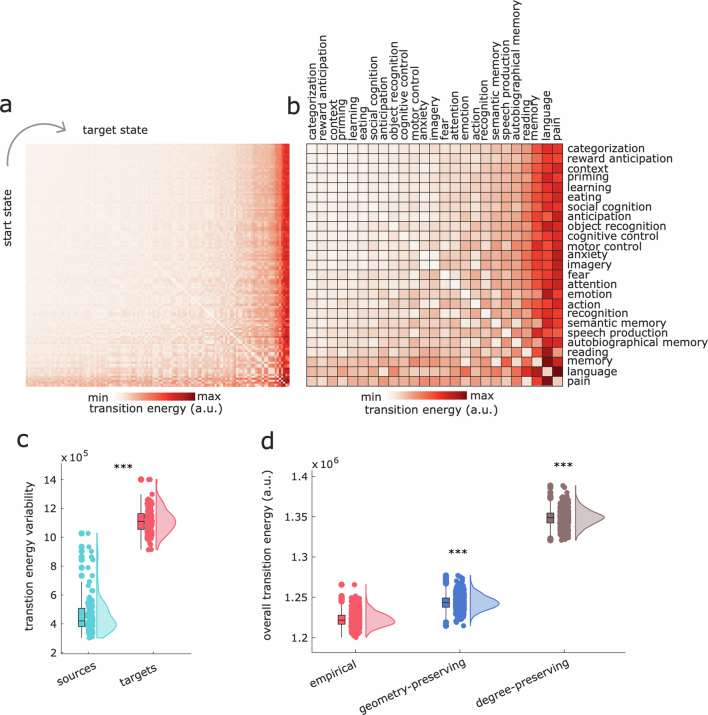
Replication with functional networks (inverse covariance) from functional MRI. (**a**) Transition cost (energy) between each pair of 123 cognitive topographies (‘states’) from NeuroSynth, using functional connectomes obtained as inverse covariance between fMRI BOLD timeseries from n = 100 HCP participants. Rows indicate source states, columns indicate target states. (**b**) Transition cost between each pair of a subset of 25 out of 123 NeuroSynth states, shown for visualisation purposes. Matrices are sorted by increasing cost across both rows and columns. (**c**) Variability (standard deviation) of transition energy is greater along the column dimension (target states) than along the row dimension (source states), for each combination of n = 123 pairs of cognitive topographies. t(244) = 37.06, p < 0.001, Cohen’s d = 4.73. Box-plots: center line, median; box limits, upper and lower quartiles; whiskers, 1.5× interquartile range; ***, p < 0.001 from independent-samples t-tests (two-sided). (**d**) Overall transition energy (averaged across all transitions between possible pairs of 123 cognitive topographies) for each of n = 100 HCP participants, based on the individual inverse covariance network, and the corresponding degree- and cost-preserving network null models. Empirical vs geometry-preserving: t(99) = 53.15, p < 0.001, Cohen’s d = 6.55. Empirical vs degree-preserving: t(99) = 89.01, p < 0.001, Cohen’s d = 12.83. Box-plots: center line, median; box limits, upper and lower quartiles; whiskers, 1.5× interquartile range; ***, p < 0.001 from paired-samples t-tests (two-sided). Source data are provided as a Source Data file. Source data

The present results are based on structural connectivity networks reconstructed using diffusion-weighted MRI; an alternative approach would be to use functional connectivity networks reconstructed using fMRI. Following the approach outlined by Scheid and colleagues^71^, we repeat our analyses using functional connectivity networks estimated using regularized inverse covariance between resting-state fMRI time series from the same 100 HCP participants. We find a similar pattern of asymmetry as for our main results, with target state being the main determinant of the energetic cost of transitions (Extended Data Fig. 9a–c). Just like with the empirical structural connectome, individuals’ empirical functional connectomes outperform both geometric and degree-preserving rewired network null models, in terms of supporting low-cost transitions (Extended Data Fig. 9d).

**Extended Data Fig. 10:**
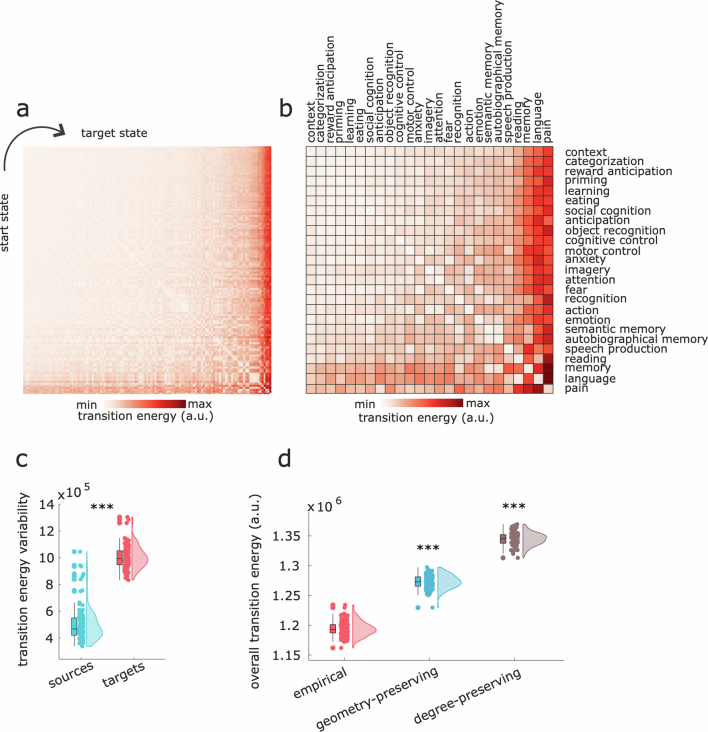
Replication in 989 HCP individuals. (**a**) Transition cost (energy) between each pair of 123 cognitive topographies (‘states’) from NeuroSynth, using the consensus connectome from n = 989 HCP individuals. Rows indicate source states, columns indicate target states. (**b**) Transition cost between each pair of a subset of 25 out of 123 NeuroSynth states, shown for visualisation purposes. Matrices are sorted by increasing cost across both rows and columns. (**c**) Variability (standard deviation) of transition energy is greater along the column dimension (target states) than along the row dimension (source states), for each combination of n = 123 cognitive topographies; t(244) = 46.39, p < 0.001, Cohen’s d = 5.89. Box-plots: center line, median; box limits, upper and lower quartiles; whiskers, 1.5× interquartile range; ***, p < 0.001 from independent-samples t-tests (two-sided). (**d**) Overall transition energy (averaged across all transitions between 123 cognitive topographies) for each individual (n = 989), based on the individual SC network, and the corresponding degree- and cost-preserving network null models. Empirical vs geometry-preserving: t(988) = 112.12, p < 0.001, Cohen’s d = 2.47. Empirical vs degree-preserving: t(988) = 306.60, p < 0.001, Cohen’s d = 13.80. Box-plots: center line, median; box limits, upper and lower quartiles; whiskers, 1.5× interquartile range; ***, p < 0.001 from paired-samples t-tests (two-sided). Source data are provided as a Source Data file. Source data

We further ensure the robustness of our results by showing that they also hold in the broader dataset of 989 HCP participants, both with a consensus connectome and at the single-participant level (Extended Data Fig. 10). Once again, we find the same pattern of asymmetries (Extended Data Fig. 10a–c). We also confirm that across individuals, transitions between cognitive topographies require less energy on the human structural connectome than on degree-preserving or degree- and cost-preserving rewired null networks (Extended Data Fig. 10d).

Next, we show that analogous results can be obtained if instead of the automated NeuroSynth meta-analytic engine, we derive 66 cognitive topographies from BrainMap, an expert-curated database of published voxel coordinates from neuroimaging studies that are significantly activated or deactivated during tasks^7,68^. As with NeuroSynth, we observe asymmetry of transitions, such that targets exhibit significantly greater variability in transition energy (mean = 8.54 × 10^4^, s.d. = 6.46 × 10^3^) than sources (mean = 4.27 × 10^4^, s.d. = 9.07 × 10^3^; *t*(130) = 31.21, *P* < 0.001, Cohen’s *d* = 5.40) (Supplementary Fig. 11). Likewise, we find that, among the BrainMap terms that have been assigned to one of the six cognitive ontology domains from ref. ^11^, memory-related terms exhibit a higher median value of asymmetry than would be expected by chance (*P* = 0.043). We also replicate the result that transitions between cognitive topographies require significantly less energy on the human connectome than on degree-preserving and degree- and cost-preserving null networks, with the geometry-preserving nulls being significantly closer to the human connectome than degree-preserving ones (all *P* < 0.001; Supplementary Fig. 11).

We further repeat our analysis using individual-level contrast maps from *N* = 989 HCP individuals performing different tasks in the scanner. Specifically, we use our network control framework to compute the transition energy between brain states defined as task-related contrasts, following the approach of ref. ^27^. The results show that asymmetry is also present for this different definition of cognitive topographies, which is individual specific and based on statistical contrasts rather than meta-analytic activation, thereby offering much greater specificity (Supplementary Fig. 12a). In line with our main results, there is significantly greater variability in transition energy across target states than across source states (Supplementary Fig. 12b). In addition, we find that language-related contrasts are the most difficult to reach (Supplementary Fig. 12a)—consistent with our model based on meta-analytic maps, which also indicated ‘language’ as one of the most difficult cognitive topographies to transition to. Finally, consistent with the results of refs. ^27^ and ^72^, we show that transitions from the subjectively easier 0-back working memory task to the subjectively more demanding 2-back working memory task are significantly more difficult than the reverse (Supplementary Fig. 12c). We further extend this result by showing that the more demanding 2-back working memory task is generally more difficult to reach, across all start states, than the 0-back working memory task (Supplementary Fig. 12d). As a final demonstration, for the working memory *N*-back task fMRI, we also computed the energy corresponding to each task-related transition in a time-resolved way, that is, between rest blocks and the subsequent task blocks, for each participant among the 100 unrelated individuals. On average, transitions from rest blocks to 0-back blocks require significantly less energy than transitions from rest blocks to 2-back blocks (Supplementary Fig. 13).

## Discussion

We investigated how the brain’s network architecture shapes its capacity to transition between behaviourally defined cognitive topographies. We also systematically modelled how transitions between cognitive topographies could be reshaped by engaging different neurotransmitter systems or by changes in cortical thickness associated with different diagnostic categories. By taking into account network structure, functional activation and chemoarchitecture, our results provide a first step towards designing interventions that selectively manipulate cognitively relevant activation.

Up to this point, a comprehensive ‘look-up table’ charting the transitions between cognitively relevant brain states has remained elusive. The present approach permits exploration of the full range of possible transitions between experimentally defined brain states with a cognitive interpretation. In this sense, our work provides a large-scale generalization of recent advances^27,54,73^ that defined brain states in terms of *β* maps from a task-based fMRI contrast, task-derived *k*-means clustering or electrocorticography signal power associated with memory task performance^31^. Importantly, we have also expanded the network control framework to include naturalistic, empirically defined forms of control input, such as of receptor density (as could be exogenously engaged by pharmacological interventions) and changes in cortical thickness (as could arise endogenously in various diagnostic categories).

We find that transitions between different cognitive topographies are not symmetric but rather directional. Namely, some cognitively relevant brain states are substantially harder to reach than others, regardless of start state. These observations are consistent with the notion that state-to-state transition cost can perhaps be framed as cognitive demand^34^: in particular, transitioning to a more cognitively demanding 2-back task requires more control energy than transition to an easier task^27,72^, as our own results also indicate. However, ‘cognitive demand’ is a multifaceted construct with a variety of distinct possible operationalizations^74^: it remains to be determined which of these different interpretations is best aligned with network control energy. This endeavour will be facilitated by the approach introduced here, which enables the computational assessment of transition costs between any number of experimentally defined tasks from the literature.

Consistent with our work, a recent study using a different operationalization of controllability reported asymmetries in the transitions between the distributions of states (defined by *k*-means clustering) observed during different tasks, including evidence that transitioning from an easier task towards a harder one requires more energy than the reverse transition^73^. In addition, a recent report identified a transition asymmetry between artificial ‘bottom-up’ and ‘top-down’ states, defined as recruiting different portions of the cytoarchitectonic sensory-fugal axis^38^, finding that top-down states (that is, involving a greater proportion of higher-order cortices) are more demanding. Indeed, we find that association of the target state with the putative unimodal–transmodal functional hierarchy is a predictor of transition cost. Moreover, the variability in ease-of-transition that we observe highlights cognitive topographies related to language and especially memory among those with the greatest asymmetry in transition cost. Both of these domains emerge gradually over human development^75,76^, and both language and autobiographical memory have long been argued (though not without controversy) to be ‘uniquely human’^77–80^. Our results of low-energy states converging on DMN topography are in line with several recent reports, which highlight that the DMN may play a role of global workspace in the human brain. By analysing dynamic connectivity across tasks and rest fMRI, Diez and Sepulcre^81^ concluded that the DMN may play an attractor-like role across cognitive states, favouring easily reachable brain configurations. Also combining task and rest fMRI data, Deco and colleagues^82^ identified posterior DMN regions (including left precuneus and left posterior cingulate) as a global workspace, based on their consistent role as targets of directed information transfer. Another approach based on information theory also identified the DMN (including prominent involvement of posterior cingulate and left angular gyrus) as a global workspace, based on its high prevalence of synergistic interactions with the rest of the brain, which are disrupted by pharmacological and pathological perturbations of consciousness^83^. Our present identification of DMN regions as a shared topography of cognitive states that are well suited to facilitate efficient transitions, therefore, converges with the emerging characterization of the DMN as orchestrating information flow and dynamics of the brain.

Although there is variability among cognitive topographies in terms of transition cost, the wiring of the human connectome generally facilitates more efficient transitions than alternative topologies^20,64,84,85^. Specifically, the human connectome enables transitions at lower cost compared with randomly rewired nulls that preserve degree sequence, suggesting that this efficiency is imparted by network topology, rather than low-level features such as the density and degree. Importantly, this efficiency can be partly attributed to the geometry of wiring lengths: when this was accounted for in our geometry-preserving null models, the connectome’s advantage was substantially diminished. Our work contributes to a growing appreciation for how network topology and geometry shape efficient communication^6,66,86,87^ and brain function^13,15,88,89^.

We also identified several factors that contribute to transition costs. Our results pertaining to Euclidean distance predicting transition costs are in line with those of Karrer and colleagues^48^ and Stiso and colleagues^31^, who found a monotonic increase of both minimum and optimal control energy with increasing distance between initial and target states. In terms of the states themselves, the best architectural predictor of transition cost to a given cognitive topography is its network-based variance (Extended Data Fig. 2). A distribution of values over a network’s nodes has high network-based variance if nodes with high values are relatively difficult to reach using a diffusion process^90^. Network control theory predicts that control energy will diffuse along the network’s paths^84^. Therefore, we can interpret our results as showing that if a state requires great activation at nodes that are difficult to reach via diffusion, the corresponding state will be harder to reach. Indeed, network-based variance is related to bidirectional communicability between nodes—a known predictor of transition energy between states^28,29,38^.

In other words, if a desired pattern of activations has low divergence from the pattern of diffusion-based proximities between nodes (low network-based variance), then that pattern will be easier to reach through network control. Finally, we systematically quantified alternative naturalistic forms of control, via neurotransmitter receptor engagement and changes in cortical morphology associated with various diagnostic categories.

We found that changes in transition energy associated with specific patterns of cortical thickness are highly heterogeneous. They are both specific to each diagnostic category and specific to each target brain state. These results should not be taken as a claim that changes in cortical thickness are the cause of a given condition. Each diagnostic category is unique and characterized by a complex set of intertwined aetiologies that may occur at different points of the lifespan. But once cortical thickness changes are in place—whatever their origin—the model quantifies the potential for these changes to facilitate cognitive transitions. Of note, we find that depression and ADHD, both of which involve widespread attentional deficits^91,92^, are characterized by overall transition costs that exceed what would be expected if the corresponding cortical thickness increases and decreases were spatially distributed in a random fashion. Other diagnostic categories (schizophrenia, bipolar and epilepsy) exhibited the opposite pattern. This suggests that localized perturbations in the connectome can attenuate some types of state transition and promote others (compared with equivalent random maps), potentially providing a mechanistic link between regional anatomical changes and changes in cognitive capacity across diagnostic categories. This observation is consistent with results in the healthy population, where increased controllability is advantageous for cognitive performance in some regions, but disadvantageous in others^30^.

Our results pertaining to diagnostic categories are complementary to applications of network control theory that evaluated transitions between random states or intrinsic connectivity networks, based on patients’ reorganized connectomes^28,39,43^—including a recent report that temporal lobe epilepsy induces deficits in control energy that are predictive of metabolic deficits quantified by FDG-PET^43^. By contrast, here we used the healthy connectome and simulated the effect of altered control input informed by changes in cortical thickness (a change in control inputs rather than controlled network) and assessed the cost of transitioning between cognitive topographies that were experimentally defined. Since diagnostic categories typically involve both regional and connectomic alterations, in the future, we expect that combining the two approaches will provide an even more fine-grained characterization of how disease reshapes the brain’s capacity to transition between brain states.

We observed a similar principle when control inputs were guided by empirically derived receptor and transporter maps. We find that the more difficult cognitive topographies to reach also appear to be those that could most benefit from engagement of specific neurotransmitter systems, such as via pharmacological intervention. Here dopamine transporters and D_1_ receptors, histamine H_3_ receptors and mu-opioid receptors appear well positioned to facilitate transitions. These results are consistent with the use of modafinil and methylphenidate as cognitive enhancers and to treat symptoms of ADHD: both drugs engage the dopaminergic system by blocking dopamine transporter as one of their main mechanisms of action^93–106^. Likewise, H_3_-receptor antagonist drugs such as pitolisant are being evaluated for potential treatment of ADHD symptoms^107–110^.

Collectively, the predictions generated by our report highlight numerous potential clinical and non-clinical applications. Although preliminary, these results provide a first step towards designing protocols that selectively promote transitions to desired cognitive topographies in specific diagnostic categories. In addition, outcomes of this computational screening could be further tested in vivo by engaging different neurotransmitter systems through targeted pharmacological manipulations^27,33^ and evaluating the degree to which they facilitate switching between specific experimentally defined cognitive topographies.

The present work should be interpreted with respect to several important methodological considerations. Network control theory models neural dynamics as noise-free, and under assumptions of linearity and time invariance^20,23,48^. Recent work has begun to introduce stochasticity in the network control framework for the brain, with promising results—though still within the context of linear systems^54,73^. Although the brain is a nonlinear system, it has been shown that nonlinear dynamics can be locally approximated by linear dynamics^17,111^, including through the application of dynamic causal models^112,113^. In fact, evidence suggests that linear models may even outperform nonlinear ones at the macroscopic scale of functional MRI signals^114,115^. Finally, the predictions of linear network control theory have found successful translation to nonlinear systems^45,116^, and even at predicting the effects of direct intracranial electrical stimulation in humans^31^.

In addition, we made several simplifying assumptions about the control input provided by each region based on its cortical thickness or receptor/transporter expression. We have treated summary statistics from NeuroSynth’s term-based meta-analysis as representing relative activation; we also acknowledge that the mapping of functional activation to psychological terms in NeuroSynth does not distinguish activations from deactivations^8^. However, we believe that our replication with cognitive topographies defined using BrainMap^68^ provides reassurance about the validity of our approach. We also repeated our analysis using individual-level contrast maps from in-scanner tasks. The resulting cognitive topographies are therefore explicit activations (as opposed to meta-analytic estimates) elicited by specific tasks, pertaining to each individual (rather than being aggregates from the literature). This analysis therefore enabled us to overcome some of the limitations inherent to NeuroSynth, providing greater specificity and further validating our computational framework and its results. This validation includes the observation that the subjectively more demanding 2-back working memory task is also energetically more difficult to reach than a comparatively easier 0-back task—in line with previous reports^27,72,73^.

Although the ENIGMA consortium provides datasets from large cohorts with standardized pipelines, ensuring robust results, the patient populations may exhibit co-morbidities and/or be undergoing treatment. In addition, and of particular relevance for the present modelling approach, the available maps do not directly reflect changes in tissue volume but rather the effect size (magnitude of between-group difference) of patient-control statistical comparisons (though note that our use of spatial autocorrelation-preserving null models accounts for the mean and variance of each map; Methods). Future work should combine assessment of cortical thickness in patient populations with task-based fMRI across several tasks, to evaluate whether the cortical thickness changes are predictive of changes in the energetic cost of different transitions. Although here we made the simplifying assumption of modelling cortical atrophy as decreased intrinsic control input, and cortical thickening as increased control input, we acknowledge that these are complex phenomena, such that enlargement could also impair regional engagement. Additional control input is also not necessarily beneficial, if it imposes energetic or other burdens. Moreover, many disorders, diseases and conditions exist beyond the diagnostic categories considered here. The same limitation applies to the PET data: the atlas of neurotransmitter receptors, though extensive, does not include all receptors. However, our computational workflow can readily be extended to accommodate new cognitive topographies, receptors, or disease maps of interest.

Pertaining to our modelling of neurotransmitter engagement, it is important to clarify that our computational framework is intended to simulate the effect of engaging a given neuromodulatory system by agonism of one of its receptors, such as a pharmacological intervention might do. This is distinct from external neuromodulation via transcranial magnetic stimulation, or transcranial direct current stimulation, or deep brain stimulation^30,31,44,45^. Even though such interventions may also be suitable for modelling via network control theory, it is not our purpose to do so here. In addition, control inputs applied to skewed receptor density distributions do not account for the downstream effects of engaging a neurotransmitter system. In addition, neurons can respond to medications, especially with chronic treatment, via the up/downregulation of receptors and transporters^117–120^. We expect that future work will benefit from complementing the tractability of linear control theory with the additional neurobiological realism offered by nonlinear computational models, such as biophysical models of coupled excitatory and inhibitory populations^121,122^. Recent work has shown that such models can be enriched with PET maps to simulate the effects of excitatory or inhibitory stimulation and recapitulate the empirical effects of pharmacological agents^123–126^. Evaluating such models’ ability to achieve distinct cognitively relevant activation patterns in response to different pharmacological perturbations will further expand our ability to characterize cognitive operations in silico and understand their computational origins.

Finally, here we did not consider the role of the subcortex, which is not present in ENIGMA cortical alteration maps, and needs different treatment both in terms of spatial null models and in terms of PET imaging. This approach has also been adopted in recent applications of network control theory to the human connectome^38,41^. In keeping with the literature on network control theory in human neuroscience, we have relied on parcellated brain data. Although we have shown that our results are robust to the use of alternative parcellations of the cerebral cortex (both functional and anatomical), future studies combining high-resolution functional and diffusion data will help describe transition energies at the voxel level.

## Outlook

Overall, our approach based on network control theory provides a computational framework to evaluate the propensity of the connectome to support transitions between cognitively relevant brain patterns. This framework lends itself to interrogating transitions between specific states of interest and modelling the impact of global perturbations of the connectome, or regional cortical heterogeneities, or specific neurotransmitter systems. We anticipate that future work may combine different facets of this approach to evaluate in silico which potential pharmacological treatments may best address the specific cognitive difficulties associated with a given disorder or brain tissue lesion.

## Methods

### Human structural connectome from HCP

We used dMRI data from the 100 unrelated participants (54 females and 46 males, mean age = 29.1 ± 3.7 years) of the HCP 900 participants’ data release^61^. All HCP scanning protocols were approved by the local Institutional Review Board at Washington University in St. Louis. The DWI acquisition protocol is covered in detail elsewhere^127^. The dMRI scan was conducted on a Siemens 3T Skyra scanner using a 2D spin-echo single-shot multiband echo-planar imaging sequence with a multiband factor of 3 and monopolar gradient pulse. The spatial resolution was 1.25 mm isotropic (repetition time (TR) = 5,500 ms, time to echo (TE) = 89.50 ms). The *b*-values were 1,000 s mm^−^^2^, 2,000 s mm^−^^2^ and 3,000 s mm^−^^2^. The total number of diffusion sampling directions was 90, 90 and 90 for each of the shells in addition to 6 b0 images. We used the version of the data made available in DSI Studio-compatible format at http://brain.labsolver.org/diffusion-mri-templates/hcp-842-hcp-1021 (ref. ^128^).

We adopted previously reported procedures to reconstruct the human connectome from DWI data. The minimally preprocessed DWI HCP data^127^ were corrected for eddy current and susceptibility artefact. DWI data were then reconstructed using q-space diffeomorphic reconstruction (QSDR)^129^, as implemented in DSI Studio (https://www.dsi-studio.labsolver.org). QSDR is a model-free method that calculates the orientational distribution of the density of diffusing water in a standard space to conserve the diffusible spins and preserve the continuity of fibre geometry for fibre tracking. QSDR first reconstructs diffusion-weighted images in native space and computes the quantitative anisotropy (QA) in each voxel. These QA values are used to warp the brain to a template QA volume in Montreal Neurological Institute (MNI) space using a nonlinear registration algorithm implemented in the statistical parametric mapping (SPM) software. A diffusion sampling length ratio of 2.5 was used, and the output resolution was 1 mm. A modified FACT algorithm^130^ was then used to perform deterministic fibre tracking on the reconstructed data, with the following parameters^131^: angular cut-off of 55°, step size of 1.0 mm, minimum length of 10 mm, maximum length of 400 mm, spin density function smoothing of 0.0 and a QA threshold determined by the DWI signal in the cerebrospinal fluid. Each of the streamlines generated was automatically screened for its termination location. A white matter mask was created by applying DSI Studio’s default anisotropy threshold (0.6 Otsu’s threshold) to the spin distribution function’s anisotropy values. The mask was used to eliminate streamlines with premature termination in the white matter region. Deterministic fibre tracking was performed until 1,000,000 streamlines were reconstructed for each individual.

For each individual, their structural connectome was reconstructed by drawing an edge between each pair of regions *i* and *j* from the Desikan-Killiany cortical atlas^132^ if there were white matter tracts connecting the corresponding brain regions end to end; edge weights were quantified as the number of streamlines connecting each pair of regions, normalized by region-of-interest distance and size.

A group-consensus matrix *A* across participants was then obtained using the distance-dependent procedure of Betzel and colleagues to mitigate concerns about inconsistencies in reconstruction of individual participants’ structural connectomes^133^. This approach seeks to preserve both the edge density and the prevalence and length distribution of inter- and intra-hemispheric edge length distribution of individual participants’ connectomes, and it is designed to produce a representative connectome^15,133^. This procedure produces a binary consensus network indicating which edges to preserve. The final edge density was 27%. The weight of each non-zero edge is then computed as the mean of the corresponding non-zero edges across participants.

### Structural connectomes from 989 HCP participants

For the replication with *N* = 989 HCP young adult participants^61^, a multi-shell, multi-tissue constrained spherical deconvolution model was computed in MRtrix3 to estimate the orientation distribution function^134^. We used a deterministic tractography algorithm^135^ with dynamic white matter seeding to create individual, whole-brain tractograms containing five million streamlines for each participant. The structural connectivity between any two regions was the number of streamlines connecting those regions divided by the sum of the grey matter volume of those regions. The result was an ROI-volume normalized pairwise structural connectivity matrix for each individual. A consensus connectome was also generated, as described above.

### Functional connectivity

We quantified functional connectivity using resting-state fMRI data from the same *N* = 100 unrelated HCP participants. Data were acquired using the following parameters. Structural MRI: 3D MPRAGE T1-weighted, TR = 2,400 ms, TE = 2.14 ms, TI = 1,000 ms, flip angle = 8°, field of view (FOV) = 224 × 224, voxel size = 0.7 mm isotropic. Two sessions of 15 min resting-state fMRI: gradient-echo EPI, TR = 720 ms, TE = 33.1 ms, flip angle = 52°, FOV = 208 × 180, voxel size = 2 mm isotropic. Here we used functional data from only the first scanning session in left-right (LR) direction. HCP-minimally preprocessed data^127^ were used for all acquisitions. The minimal preprocessing pipeline includes bias field correction, functional realignment, motion correction and spatial normalization to MNI (MNI-152) standard space with 2 mm isotropic resampling resolution^127^. We also removed the first 10 volumes to allow magnetization to reach steady state. Additional denoising steps were performed using the SPM12-based toolbox CONN (http://www.nitrc.org/projects/conn), version 17f (ref. ^136^). To reduce noise owing to cardiac and motion artefacts, we applied the anatomical CompCor method of denoising the functional data. The anatomical CompCor method (also implemented within the CONN toolbox) involves regressing out of the functional data the following confounding effects: the first five principal components attributable to each individual’s white matter signal and the first five components attributable to the individual cerebrospinal fluid signal; six subject-specific realignment parameters (three translations and three rotations) as well as their first-order temporal derivatives^137^. Linear detrending was also applied, and the subject-specific denoised BOLD signal time series were band-pass filtered to eliminate both low-frequency drift effects and high-frequency noise, thus retaining frequencies between 0.008 Hz and 0.09 Hz. Subsequently, following ref. ^71^, functional connectivity between pairs of regional BOLD time series was estimated for each participant as the regularized inverse covariance matrix. Negative values were removed, and a consensus functional connectome was obtained as the mean across individual participants’ functional connectomes.

### Alternative structural connectome from Lausanne dataset

A total of *N* = 70 healthy participants (25 females, age 28.8 ± 8.9 years old) were scanned at the Lausanne University Hospital in a 3 Tesla MRI scanner (Trio, Siemens Medical) using a 32-channel head coil^138^. Informed written consent was obtained for all participants in accordance with institutional guidelines, and the protocol was approved by the Ethics Committee of Clinical Research of the Faculty of Biology and Medicine, University of Lausanne. The protocol included (1) a magnetization-prepared rapid acquisition gradient echo (MPRAGE) sequence sensitive to white/grey matter contrast (1 mm in-plane resolution, 1.2 mm slice thickness) and (2) a DSI sequence (128 diffusion-weighted volumes and a single b0 volume, maximum *b*-value 8,000 s mm^−^^2^, 2.2 × 2.2 × 3.0 mm voxel size).

Structural connectomes were reconstructed for individual participants using deterministic streamline tractography and divided according to the Desikan-Killiany grey matter parcellation. White matter and grey matter were segmented from the MPRAGE volumes using the FreeSurfer version 5.0.0 open-source package, whereas DSI data preprocessing was implemented with tools from the Connectome Mapper open-source software, initiating 32 streamline propagations per diffusion direction for each white matter voxel. Structural connectivity was defined as streamline density between node pairs, that is, the number of streamlines between two regions normalized by the mean length of the streamlines and the mean surface area of the regions, following previous work with these data^69,139^.

### Network control energy

Network control theory models the brain as a linear, time-invariant control system^23^. In the general context of linear control theory, the evolutionary dynamics of the state *x*(*t*) is formulated as an equation relating the first-order derivative of the state *x*(*t*), $\dot{x}$, to the state variable *x* itself and the control input. For this system, given the initial and target states, the control trajectory moving from the initial to target states is determined by the interaction matrix *A*, the input matrix *B* and the control input *u*(*t*) in the form of

1 $$\dot{x}={Ax}(t)+{Bu}(t)$$

The state interaction matrix *A* characterizes the relationships between system elements, determining how the control system moves from the current state to the future state. The structural connectivity matrix *A* serves as a linear operator that maps each state, *x*, to the rate of change of that state. This linear transformation can be described in terms of the evolutionary modes of the system consisting of the *N* eigenvectors of *A* and their associated eigenvalues. The matrix *A* is then normalized to avoid infinite growth of the system over time:

2 $${A}_{\text{norm}}=\frac{A}{{\rm{|}}\lambda (A{)}_{\max }{\rm{|}}+c}-I$$

Here *I* denotes the identity matrix of size *N* × *N*, and ${\rm{|}}\lambda (A{)}_{\max }{\rm{|}}$ denotes the largest eigenvalue of the system. To normalize the system, we must specify the parameter *c*, which determines the rate of stabilization of the system. Here we use *c* = 0 for our main analyses, such that the system approaches its largest mode over time. We also report results for $c=0.01\times {\rm{|}}\lambda (A{)}_{\max }{\rm{|}}$, whereby all modes decay, and the system goes to zero over time. The control input matrix *B* denotes the location of control nodes on which we place the input energy. If we control all brain regions, *B* corresponds to the *N* × *N* identity matrix with ones on the diagonal and zeros elsewhere. If we control only a single brain region *i*, *B* reduces to a single *N* × *N* diagonal matrix with a one in the *i*th element of the diagonal and zeros elsewhere. The control input *u*(*t*) denotes the amount of energy injected into each control node at each time point *t*. Intuitively, *u*(*t*) can be summarized over time to represent the total energy consumption during transition from an initial state to a final state. In the brain control analysis framework, a state refers to a vector *x*(*t*) of *N* elements, which encodes the neurophysiological activity map across the whole brain. In the current work, *x*(*t*) is the meta-analytic activation of each region associated with each cognitively relevant term, aggregated over studies in the NeuroSynth database.

This computational approach allows us to compute the transition energy as the optimal energy required to transition between each pair of cognitive topographies in finite time.

To explore the energetic efficiency of the structural brain network in facilitating the transition between cognitive topographies, we adopted the optimal control framework to estimate the control energy required to optimally steer the brain through these state transitions^28,29,43^. Optimality is defined in terms of jointly minimizing the combination of both the length of the transition trajectory from an initial source state $(x(0)={x}_{0})$ to the final target state $(x(T)={x}_{T})$ over the time horizon *T* (to avoid spurious, unrealistically long trajectories) and the required unique control input ${u}^{* }(t)$ summarized over the length of this trajectory:

3 $$\begin{array}{rcl}u{(t)}_{\kappa }^{\ast } & = & \mathop{\mathrm{argmin}}\limits_{u_\kappa }J({u}_{\kappa })\\ & = & \mathop{\mathrm{argmin}}\limits_{u_\kappa }{\displaystyle\int }_{0}^{T}\left(\right.({x}_{T}-x(t))^{\top}({x}_{T}-x(t))\\ & + & \rho {u}_{\kappa }(t)^{\top}{u}_{\kappa }(t)\left.\right)dt\end{array}$$

where $({x}_{T}-x(t))^{\top}({x}_{T}-x(t))$ is the distance between the state at time *t* and the final state *x*_*T*_, *T* is the finite amount of time given to reach the final state, and *ρ* is the relative weighting between the cost associated with the length of the transition trajectory and the input control energy. The equation is solved using forward integration. $J(u(t{)}_{\kappa }^{* })$ is the cost function defined to find the unique optimal control input $u(t{)}_{\kappa }^{* }$. Here, following common practice, we set *ρ* equal to 1, corresponding to equal weighting^28,48^. We set *T* = 1 (ref. ^28^), but we also report results with *T* = 3 (ref. ^48^) (Extended Data Figs. 3–6). Note that transitions between states are not necessarily symmetric in terms of energy required. Asymmetric transitions are possible when considering network control energy because of the system’s dynamics: activity on the network evolves spontaneously even in the absence of control inputs as a diffusion process. When a transition is in accordance with this spontaneous evolution, it will be easier than when it opposes it. This is analogous to how it takes less effort to move with the current of a river than against it.

### Cognitive topographies from NeuroSynth

Continuous measures of the association between voxels and cognitive categories were obtained from NeuroSynth, an automated term-based meta-analytic tool that synthesizes results from more than 14,000 published fMRI studies by searching for high-frequency keywords (such as ‘pain’ and ‘attention’ terms) that are systematically mentioned in the papers alongside fMRI voxel coordinates (https://github.com/neurosynth/neurosynth, using the volumetric association test maps)^8^. This measure of association strength is the tendency that a given term is reported in the functional neuroimaging study if there is activation observed at a given voxel. Note that NeuroSynth does not distinguish between areas that are activated or deactivated in relation to the term of interest, nor the degree of activation, only that certain brain areas are frequently reported in conjunction with certain words. Unlike BrainMap^7,68^, which is manually curated by experts, NeuroSynth is an automated tool and therefore includes a broader set of terms. Although more than a thousand terms are catalogued in the NeuroSynth engine, we refine our analysis by focusing on cognitive function and therefore we limit the terms of interest to cognitive and behavioural terms. To avoid introducing a selection bias, we opted for selecting terms in a data-driven fashion instead of selecting terms manually. Therefore, terms were selected from the Cognitive Atlas, a public ontology of cognitive science^62^, which includes a comprehensive list of neurocognitive terms. This approach totalled to *t* = 123 terms (Supplementary Table 6), ranging from umbrella terms (‘attention’ and ‘emotion’) to specific cognitive processes (‘visual attention’ and ‘episodic memory’), behaviours (‘eating’ and ‘sleep’) and emotional states (‘fear’ and ‘anxiety’) (Supplementary Table 7) (note that the 123 term-based meta-analytic maps from NeuroSynth do not explicitly exclude patient studies). The Cognitive Atlas subdivision has previously been used in conjunction with NeuroSynth^67,140,141^, so we opted for the same approach to make our results comparable to previous reports. The probabilistic measure reported by NeuroSynth can be interpreted as a quantitative representation of how regional fluctuations in activity are related to psychological processes.

### Alternative cognitive topographies from BrainMap

Whereas NeuroSynth is an automated tool, BrainMap is an expert-curated repository: it includes the brain coordinates that are significantly activated during thousands of different experiments from published neuroimaging studies^7,68^. As a result, NeuroSynth terms and BrainMap behavioural domains differ considerably. Here we used maps pertaining to 66 unique behavioural domains (Supplementary Table 8; the same as in ref. ^141^) obtained from 8,703 experiments. Experiments conducted on unhealthy participants were excluded, as well as experiments without a defined behavioural domain.

### Cognitive topographies from HCP in-scanner tasks

As a further validation, we also adopted the approach of ref. ^27^ to define cognitive topographies based on task-related parametric contrast maps. We used the publicly released task-fMRI general linear model (GLM) outputs from the 989 HCP young adult dataset to obtain individual-level brain maps corresponding to various cognitive states and conditions (although note that not every individual had usable data for each task, whether owing to issues with acquisition or data quality). Data were acquired with the same parameters and instruments as the HCP resting-state fMRI data discussed above. Full details of the acquisition can be found in ref. ^142^. Data were first minimally preprocessed using FSL and FreeSurfer tools, which included gradient unwarping, motion correction, fieldmap-based EPI distortion correction, brain-boundary-based registration of EPI to structural T1-weighted scan, nonlinear (FNIRT) registration into MNI152 space, grand-mean intensity normalization and smoothing (2 mm full-width half at maximum kernel). Full processing details are available in ref. ^127^. Fixed-effects analyses were conducted using FSL’s fMRI Expert Analysis Tool (FEAT)^143^ to estimate the average effects across runs within participants in standard grayordinates space for each task condition. For each task, predictors were included in the model for each type of stimulus (condition) and linear contrasts of parameter estimates were computed to compare each condition to baseline and to each other. Full details of the GLM model and task designs are found in ref. ^144^.

Here we used select conditions from each task to represent individual-level states analogous to our meta-analytical approach using NeuroSynth in the main text. Namely, we used individual-level contrasts of parameter estimates (COPES) for the following task conditions compared to baseline: working memory (that is, *N*-back task; 2-back and 0-back contrasts)^145^, incentive processing (that is, gambling task; punish and reward contrasts)^146^, motor task (average of all movement conditions)^147^, language processing (math task and story task contrasts)^148^, social cognition (social and random contrasts; in this task, participants watch 20 s videos of objects either interacting (social condition) or moving randomly (random condition))^149^, relational processing (relational and matching contrasts; in this task, the relational processing condition involves identifying what dimension a pair of objects differs along (shape or texture) and then deciding whether a second pair of objects differs along the same dimension; in the matching (control) condition, participants are presented with a pair of objects and a word (shape or texture) and must decide whether a third object matches either of the first two on that dimension)^150^ and emotional processing (faces and shapes contrasts; participants are asked either which of the two faces at the top of the screen matches the face at the bottom of the screen or which of the two shapes matches the shape at the bottom of the screen; the faces are either angry or fearful in their expression)^151^. These publicly available outputs were then parcellated into 68 cortical ROIs from the Desikan-Killiany atlas^132^, producing a 1 × 68 state vector for each of these conditions for each individual. The transition energy between each pair of cognitive topographies defined in this way was then computed, for each individual, in the same way as for the NeuroSynth-derived cognitive topographies. For the analysis of time-resolved transitions in the *N*-back working memory task-fMRI HCP data, for each of the 100 unrelated individuals, the brain pattern corresponding to each state was defined by contrasting the mean activity of the corresponding task block (0-back or 2-back) and the mean activity across all ‘rest’ blocks.

### Network null models

We used two different network null models to disambiguate the role of connectome topology and geometric embedding in shaping control energy^64^. The first null model is the well-known Maslov–Sneppen degree-preserving rewired network, whereby edges are swapped to randomize the topology while preserving the exact binary degree of each node (degree sequence), and the overall distribution of edge weights^65^. As a second, more stringent null model, we adopted a null model that, in addition to preserving exactly the same degree sequence and exactly the same edge weight distribution as the original network, also approximately preserves the original network’s edge length distribution (based on Euclidean distance between regions) and the weight–length relationship^66^.

For each null model, we generated a population of 500 null networks starting from the empirical connectome and computed the control energy between each pair of cognitive brain states from NeuroSynth, as done for the empirical connectome. We compared the overall control energy between all possible states obtained from the empirical connectome and from the distribution of null instances.

### Spatial null models

To evaluate the role of regional neuroanatomical features, we implemented a permutation-based null model, termed spin test^64,67^. For each map, parcel coordinates were projected onto the spherical surface and then randomly rotated and original parcels were reassigned the value of the closest rotated parcel (10,000 repetitions)^69^. In addition to preserving the distribution of cortical values, this null model also preserves the spatial autocorrelation present in the data.

### Predictors of transition energy

We characterized each cognitive brain state from NeuroSynth in terms of its relationship with several well-known graph-theoretic properties of the structural connectome. From the consensus connectome, we computed the binary and weighted degree (also known as node strength) of each region. We also computed the participation coefficient of each region, based on the modular assignment of each region to the well-known intrinsic connectivity networks^51^. We computed the Spearman correlation between each of these vectors and each of the 123 brain maps from NeuroSynth. In addition, we also computed the correlation between each NeuroSynth map and the principal gradient of variation in functional connectivity^152^, believed to reflect the hierarchical organization of cortical information processing.

### Network-based variance

For each NeuroSynth map, we also computed as an additional predictor a recently developed measure termed ‘network variance’^90^. The traditional notion of variance of a distribution (sum of squared differences from the mean) assumes that observations are independent. However, this assumption is almost invariably violated in the case of distributions on a graph, where the graph’s nodes (whose values correspond to the distribution’s observations) are connected to each other, generating dependencies. The notion of spatial autocorrelation^67^ can be cast as a special case of this situation, whereby the graph connecting nodes is the graph of spatial distances between them (for example, Euclidean distance). Devriendt and colleagues provided the network variance as a generalization of variance to distributions on a graph:

4 $$\mathrm{var}(\,p)=\frac{1}{2}\mathop{\sum }\limits_{i,\;j}^{N}p(i)p(j){d}^{\,2}$$

In other words, a distribution on a graph has high network variance if most of the mass (here activity) is concentrated at nodes that are poorly connected with the rest of the network. This relies on defining a suitable measure of distance on a graph. Devriendt and colleagues noted that the geodesic distance (length of the shortest path between two nodes) may be a suitable candidate, but recommended using the effective resistance instead^90,153^. Like geodesic distance, the effective resistance is predicated on the length of the paths between a pair of nodes. However, unlike the geodesic distance, effective resistance does not only consider the shortest path between two nodes, but rather it takes into account paths of all lengths along the graph, such that two nodes are less distant the more paths exist between them, thereby reflecting the full topology of the network. The resistance distance *ω*_*ij*_ between nodes *i* and *j* is large when nodes *i* and *j* are not well connected in the network, such that only few, long paths connect them, resulting in a long time for a random walker to reach one node from another, whereas a small *ω*_*ij*_ means that they are well connected through many, predominantly short paths *i* and *j* (ref. ^153^). Up to a constant, the effective resistance can be computed as the ‘commute time’: the mean time it takes a random walker to go from node *i* to node *j* and back, for all pairs of nodes *i* and *j* (ref. ^154^). Concretely, effective resistance on a graph is computed as

5 $${\omega }_{ij}={({e}_{i}-{e}_{j})}^{\top}Q({e}_{i}-{e}_{j})$$

where the unit vectors have entries (*e*_*i*_)*k* *=* 1 if *k* = *i* and zero otherwise, and where *Q* is the Moore–Penrose pseudoinverse of the graph’s Laplacian matrix.

For each NeuroSynth map, we computed its network variance using as distance measure the effective resistance on the consensus connectome. Since the network variance requires the distribution on the graph’s nodes to be positive and sum to 1, each map’s values were rescaled so that the minimum was 0, and then divided by their sum. We then used these characterizations of the NeuroSynth maps (correlation with connectome graph-theoretic properties, correlation with the cortical hierarchy and network variance) as predictors against the average energy required to transition to each cognitive brain state. We performed multiple partial correlations using each characterization in turn as predictor (after partialling out the effects of mean and traditional variance of each NeuroSynth map).

### Dominance analysis

As an alternative approach, to consider all predictors together and evaluate their respective contributions, we performed a dominance analysis with all five predictors. Dominance analysis seeks to determine the relative contribution (‘dominance’ of each independent variable to the overall fit (adjusted *R*^2^)) of the multiple linear regression model (https://github.com/dominance-analysis/dominance-analysis)^155^. This is done by fitting the same regression model on every combination of predictors (2^*p*^-1 submodels for a model with *p* predictors). Total dominance is defined as the average of the relative increase in *R*^2^ when adding a single predictor of interest to a submodel, across all 2^*p*^-1 submodels. The sum of the dominance of all input variables is equal to the total adjusted *R*^2^ of the complete model, making the percentage of relative importance an intuitive method that partitions the total effect size across predictors. Therefore, unlike other methods of assessing predictor importance, such as methods based on regression coefficients or univariate correlations, dominance analysis accounts for predictor–predictor interactions and is interpretable.

### Cortical thickness and cerebral blood flow maps

We used the *neuromaps* toolbox (https://netneurolab.github.io/neuromaps/)^156^ to fetch the map of cerebral blood flow from ref. ^63^ and the map of cortical thickness from ref. ^157^.

### Patterns of cortical thickness change from the ENIGMA database

Spatial maps of case versus control cortical thickness were obtained by including all the neurological, neurodevelopmental and psychiatric diagnostic categories available from the ENIGMA (Enhancing Neuroimaging Genetics through Meta-Analysis) consortium^56,57^ and the ENIGMA Toolbox (https://github.com/MICA-MNI/ENIGMA)^55^ and recent related publications (https://github.com/netneurolab/hansen_crossdisorder_vulnerability)^58^, except for obesity and schizotypy. This resulted in a total of 11 maps, pertaining to 22q11.2 deletion syndrome^158^, attention deficit hyperactivity disorder^159^, autism spectrum disorder^160^, idiopathic generalized epilepsy^161^, right temporal lobe epilepsy^161^, left temporal lobe epilepsy^161^, depression^162^, obsessive-compulsive disorder^163^, schizophrenia^164^, bipolar disorder^165^ and Parkinson’s disease^166^. The ENIGMA consortium is a data-sharing initiative that relies on standardized image acquisition and processing pipelines, such that cortical thickness maps are comparable^57^. Altogether, over 17,000 patients were scanned across the 11 diagnostic categories against almost 22,000 controls. The values for each map are *z*-scored effect sizes (Cohen’s *d*) of cortical thickness in patient populations versus healthy controls. Imaging and processing protocols can be found at http://enigma.ini.usc.edu/protocols/.

For every brain region, we constructed an 11-element vector of cortical thickness changes, where each element represents a diagnostic category’s change in cortical thickness at the region. These values were then added to the *B* matrix of uniform control inputs to provide regional heterogeneity. This approach is motivated by the expectation that regions with decreased thickness should have lower capacity to exert control inputs, and vice versa. Recent work adopted a similar approach to model the regional control input provided at each region, in terms of the regional density of receptor expression, such that regions expressing the receptor to a greater extent are understood to exert greater control input^33^. The largest increase in cortical thickness across all diagnostic categories is 0.87 (expressed in terms of Cohen’s *d*), whereas the largest decrease is 0.59. Thus, across all diagnostic categories, the entries in the input matrix *B* were always positive, bound between 0.41 and 1.87. Since the distributions of cortical thickness increases and decreases associated with the various ENIGMA diagnostic categories are different, changing the distribution of control inputs also changes the overall amount of control energy that is being injected into the system (in some cases leading to an overall increase or an overall decrease) and consequently the control energy that is required to transition between brain states. Therefore, an appropriate null model to evaluate the effects of patterns of grey matter change associated with diagnostic categories on brain state transitions should preserve the overall spatial distribution of control inputs associated with each map while changing the spatial location. Rather than simply randomizing the distribution of cortical thickness increases and decreases, we opted to adopt the spin-based null model, which also preserves the spatial autocorrelation present in the data^64,67^. We then obtained the control energy required to transition between each pair of states (here considering only the reduced set of 25 states, to reduce computational burden) for the empirical ENIGMA map against the distribution of spin-null maps. This distribution was used to assess whether the transition cost associated with the empirical pattern of cortical thickness changes associated with each diagnostic category is more energetically demanding than would be expected if the same changes were occurring at random on the cortex (but with equivalent spatial autocorrelation).

### Receptor maps from PET

Receptor densities were estimated using PET tracer studies for a total of 18 receptors and transporters, across 9 neurotransmitter systems, recently made available by Hansen and colleagues at https://github.com/netneurolab/hansen_receptors (ref. ^60^). These include dopamine (D_1_ (ref. ^167^), D_2_ (refs. ^168–171^), DAT (ref. ^172^)), noradrenaline (NAT; refs. ^173–176^), serotonin (5-HT_1A_ (ref. ^177^), 5-HT_1B_ (refs. ^177–182^), 5-HT_2A_ (ref. ^183^), 5-HT_4_ (ref. ^183^), 5-HT_6_ (refs. ^184,185^), 5-HTT (ref. ^183^)), acetylcholine (α_4_β_2_ (refs. ^186,187^), M_1_ (ref. ^188^), VAChT (refs. ^189,190^)), glutamate (mGluR_5_ (refs. ^191,192^), NMDA (refs. ^193,194^)), GABA (GABA_A_; ref. ^195^), histamine (H_3_; ref. ^196^), cannabinoid (CB_1_; refs. ^197–200^) and opioid (MOR; ref. ^201^). Volumetric PET images were registered to the MNI-ICBM 152 nonlinear 2009 (version c, asymmetric) template, averaged across participants within each study, and then parcellated and receptors/transporters with more than one mean image of the same tracer (5-HT_1__B_, D_2_, VAChT) were combined using a weighted average^60^.

For the control energy analysis, each PET map was scaled between 0 and 1, and its regional values were added to the *B* matrix, following recent work^33^. Since the PET distributions are different, changing the distribution of control inputs also changes the overall amount of control energy that is being injected into the system and consequently the control energy that is required to transition between brain states. Therefore, an appropriate null model to evaluate which receptors are especially well poised to facilitate brain state transitions, in terms of their spatial location, should preserve the overall distribution of control inputs associated with each receptor while changing the spatial location. Rather than simply randomizing the distribution of receptor densities, we opted to adopt the more stringent spin test null^64,67^. In addition to preserving the distribution of regional receptor densities, this null model also preserves the spatial autocorrelation present in the data. We then evaluated how often (as a percentage out of all start states) a transition between two given brain states was found to require significantly less control energy when using an empirical receptor map to determine the control inputs than when using spin-randomized versions of the same map. Owing to the computationally intensive nature of this procedure (10,000 repetitions for each of the 18 PET maps), we only considered transitions between the reduced set of 25 brain states instead of the whole 123.

### Statistical analyses

Network null models and spatial autocorrelation-preserving null models were implemented as described in the preceding sections. The statistical significance of differences between transition energies was determined with non-parametric permutation *t*-tests, with 10,000 permutations. The use of non-parametric tests alleviated the need to assume normality of data distributions (which was not formally tested). All tests were two-sided, with an *α* value of 0.05. The effect sizes were estimated using Cohen’s measure of the standardized mean difference, *d*. To ensure robustness to possible outliers, correlations were quantified using Spearman’s rank-based non-parametric correlation coefficient.

### Reporting summary

Further information on research design is available in the Nature Portfolio Reporting Summary linked to this article.

## Supplementary information

Supplementary Information Supplementary figures and tables.

Reporting Summary

## Source data

Source Data for Figs. 2–5 and Extended Data Figs. 1–10 Source data.

## Data Availability

NeuroSynth meta-analytic maps are freely available from the NeuroSynth database at https://github.com/neurosynth/neurosynth. Human Connectome Project Young Adult resting-state, task-based and diffusion MRI data are available from https://www.humanconnectome.org/study/hcp-young-adult. Diffusion MRI data for the Human Connectome Project in DSI Studio-compatible format are available at http://brain.labsolver.org/diffusion-mri-templates/hcp-842-hcp-1021. The Lausanne structural connectivity dataset is available at 10.5281/zenodo.2872623. The ENIGMA cortical thickness data are provided as part of the ENIGMA Toolbox (v1.1.3), available at https://github.com/MICA-MNI/ENIGMA. PET receptor and transporter maps are available at https://github.com/netneurolab/hansen_receptors. Healthy cortical thickness and cerebral blood flow maps are available from the *neuromaps* toolbox at https://netneurolab.github.io/neuromaps (ref. ^156^). Source data are provided with this paper.

## Code availability

Code for control energy computation in MATLAB (version 2019a was used) is available at https://github.com/gushiapi/Dynamic-Trajectory. The Brain Connectivity Toolbox used for graph-theoretic properties and to generate degree-preserving null models is freely available at https://sites.google.com/site/bctnet. MATLAB code used to generate geometry-preserving null networks is freely available at https://www.brainnetworkslab.com/coderesources. The code for spin-based permutation testing of cortical correlations is freely available at https://github.com/frantisekvasa/rotate_parcellation. Third-party Python software (version 3.8 was used) for dominance analysis is freely available at https://github.com/dominance-analysis/dominance-analysis. Third-party Python software for computing the network variance is available at https://github.com/rlambiot/variance. DSI Studio code for tractography is freely available at https://dsi-studio.labsolver.org/. MRtrix3 software for tractography is freely available at https://www.mrtrix.org/download/. The CONN toolbox (v17f) for fMRI preprocessing is freely available at http://www.nitrc.org/projects/conn. The ENIGMA Toolbox (v1.1.3) for fetching disorder-related maps is freely available at https://github.com/MICA-MNI/ENIGMA. The Neuromaps toolbox for fetching brain maps (version 0.0.1) is freely available at https://netneurolab.github.io/neuromaps/. We have made code available online at https://github.com/netneurolab/luppi-neurosynth-control.git.
