# Supplementary material for: Contributions of network structure, chemoarchitecture and diagnostic categories to transitions between cognitive topographies
Source: Nat Biomed Eng. 2024 Aug 5;8(9):1142–61. doi: 10.1038/s41551-024-01242-2 (PMC11410673; doi:10.1038/s41551-024-01242-2)
Supplement: Supplementary file 1 — Supplementary figures and tables. [file 41551_2024_1242_MOESM1_ESM.pdf]

nature biomedical engineering

Supplementary information

https://doi.org/10.1038/s41551-024-01242-2

# Contributions of network structure, chemoarchitecture and diagnostic categories to transitions between cognitive topographies

In the format provided by the authors and unedited

**Contents**

Supplementary figures 1–13
Supplementary tables 1–8

## Supplementary figures

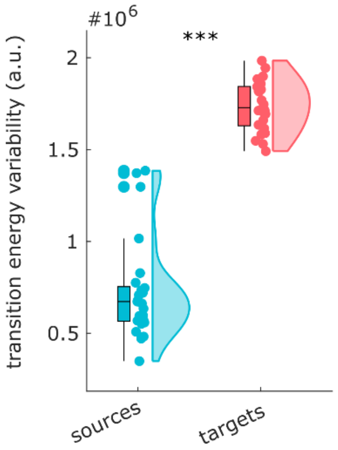


**Supplementary Fig. 1 | The 25-term subset reflects asymmetry of transitions.** Variability (standard deviation) of transition energy is greater along the column dimension (target states) than along the row dimension (source states), for the reduced set of n=25 brain states reported in Fig.2b; $t(48) = 16.02$, $p < 0.001$, Cohen's $d = 4.46$ from paired-samples t-test (two-sided). Box-plots: center line, median; box limits, upper and lower quartiles; whiskers, 1.5× interquartile range; ***, $p < 0.001$.

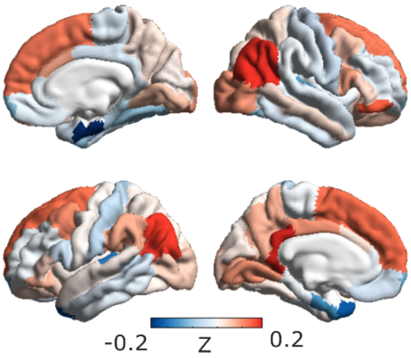


**Supplementary Fig. 2 | Cortical topography of high-centrality states in the network of transition energies |** Surface projection of the centroid (median) of cortical maps corresponding to cognitive topographies with non-zero betweenness centrality in the network of transitions.

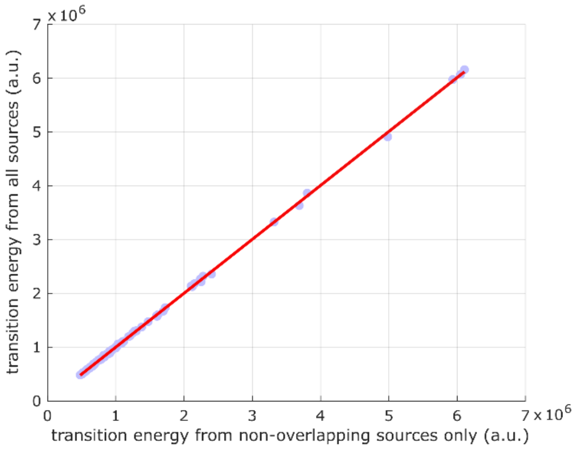


**Supplementary Fig. 3 | Removing nested and overlapping NeuroSynth terms does not alter the ranking of cognitive topographies' difficulty to reach.** Correlation between transition energy to the cognitive topographies pertaining to non-overlapping terms, from all terms (ordinate), or from non-overlapping terms only (abscissa).

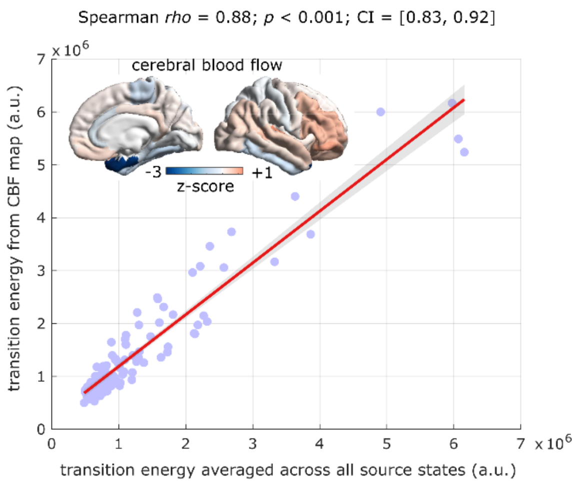


**Supplementary Fig. 4 | Transition costs between cognitive topographies recapitulate transition costs from baseline.** Spearman correlation (two-sided) between average transition energy starting from n=123 cognitive topographies (abscissa) and starting from a map corresponding to z-scored regional cerebral blood flow (CBF, shown plotted on the cortical surface).

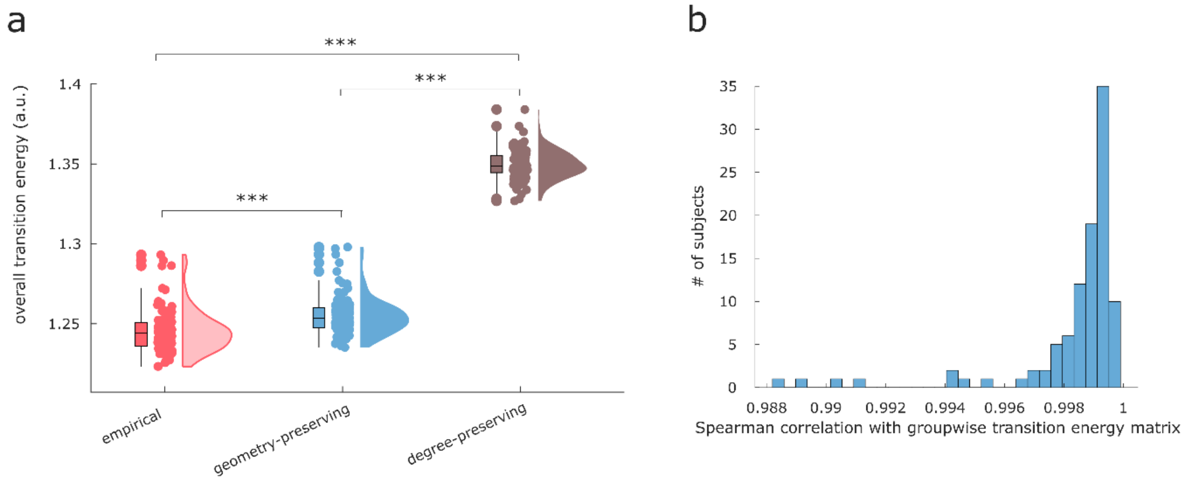


**Supplementary Fig. 5 | Replication at the single-participant level. (a)** Overall transition energy (averaged across all transitions), for each of n=100 participants and for the corresponding degree- and cost-preserving nulls. Box-plots: center line, median; box limits, upper and lower quartiles; whiskers, 1.5× interquartile range; ***, $p < 0.001$ from paired-samples t-tests (two-sided). **(b)** Histogram of the correlation between each participant's matrix of transition energies, and the matrix of transition energies obtained from the group-wise consensus structural connectome.

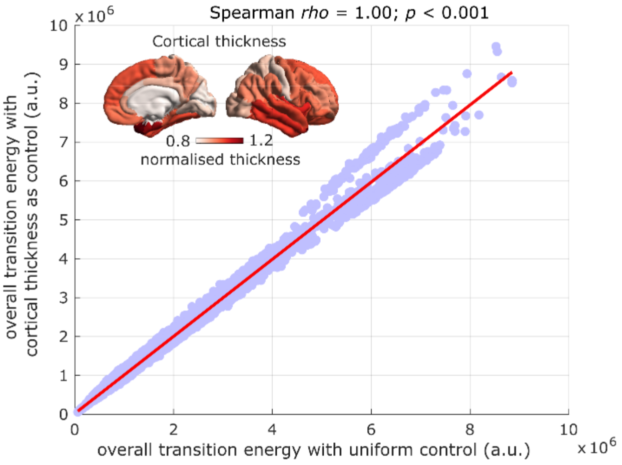


**Supplementary Fig. 6 | Transition energy using uniform versus regionally heterogenous control inputs based on regional cortical thickness.** Spearman correlation (two-sided) between overall transition energy between all pairs of n=123 cognitive topographies using uniform control inputs at each region (abscissa) and using control inputs proportional to the regional cortical thickness (shown plotted on the surface), scaled to have unit mean (ordinate).

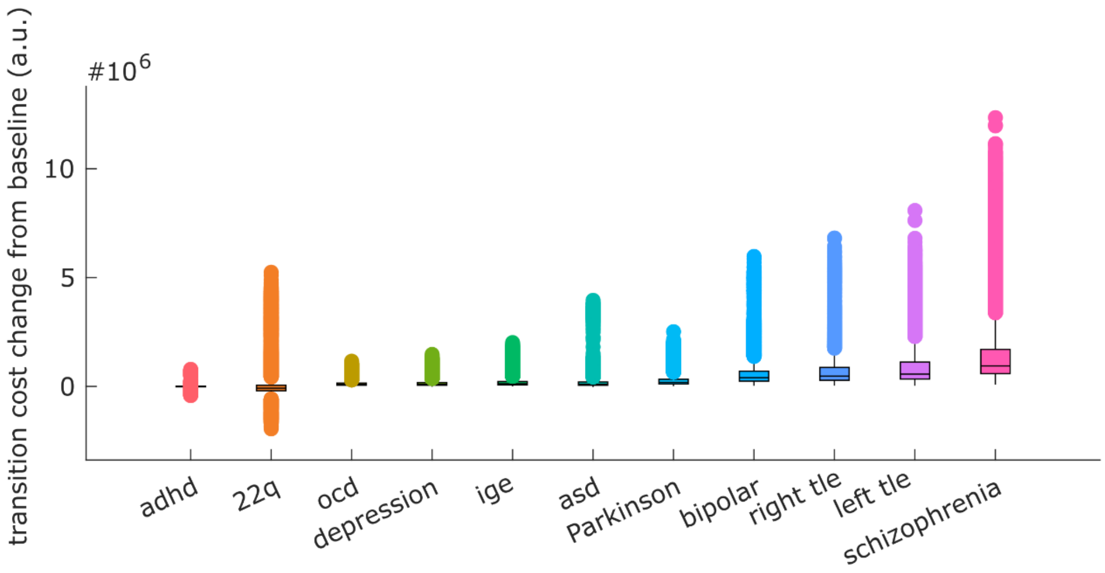


**Supplementary Fig. 7 | Overall transition energy when applying control inputs according to maps of cortical thickness change.** Each data-point represents the energy to transition to one target state (n=123), averaging across all source states. Boxplots: center line, median; box limits, upper and lower quartiles; whiskers, 1.5× interquartile range; points, outliers.

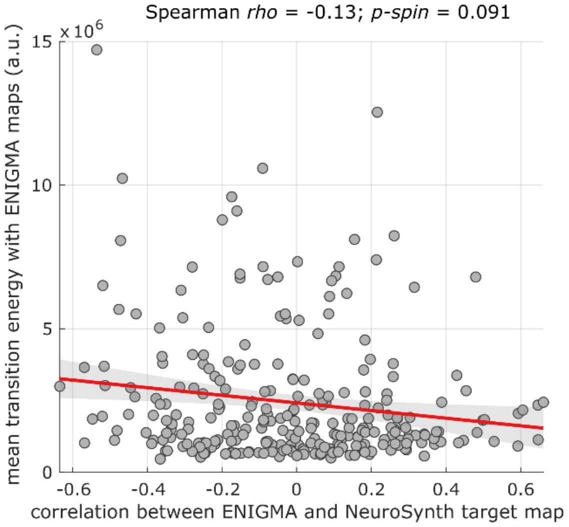


**Supplementary Fig. 8 | Spatial correspondence between ENIGMA maps and NeuroSynth maps does not correlate with transition energy.** Abscissa: spatial correlation between each ENIGMA map, and the NeuroSynth map associated with each cognitive topography from the subset of 25 used for Fig.4. Ordinate: energy to reach a given cognitive topography in the subset of 25 (averaged across all starting topographies from the same subset) using a given ENIGMA map as input. Correlation between the two distributions is not significant against a null distribution of correlations obtained from 1,000 spatial autocorrelation-preserving rotations of the NeuroSynth maps.

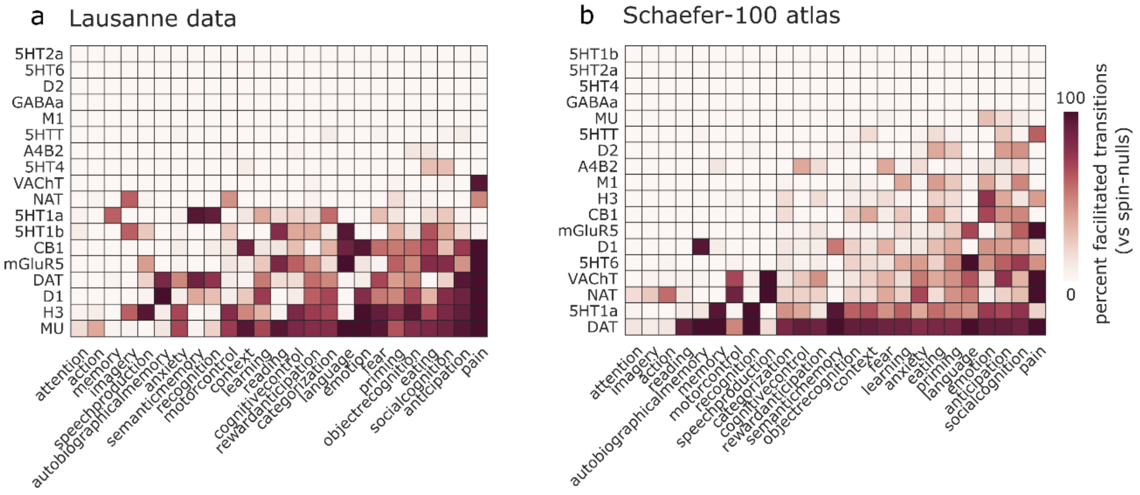


**Supplementary Fig. 9 | How neurotransmitter systems can reshape the energy landscape of the human brain, for alternative connectome dataset and alternative cortical parcellation.** Heatmaps show how each receptor/transporter reshapes the average cost of reaching a given cognitive brain state from all other states, as a percentage of transitions to each state that are facilitated, when compared against a null distribution of randomly rotated maps with preserved spatial autocorrelation and the same receptor/transporter density levels, but occurring at different neuroanatomical locations. **(a)** For the Lausanne DSI dataset; **(b)** for Human Connectome Project data parcellated using the Schaefer-100 cortical atlas.

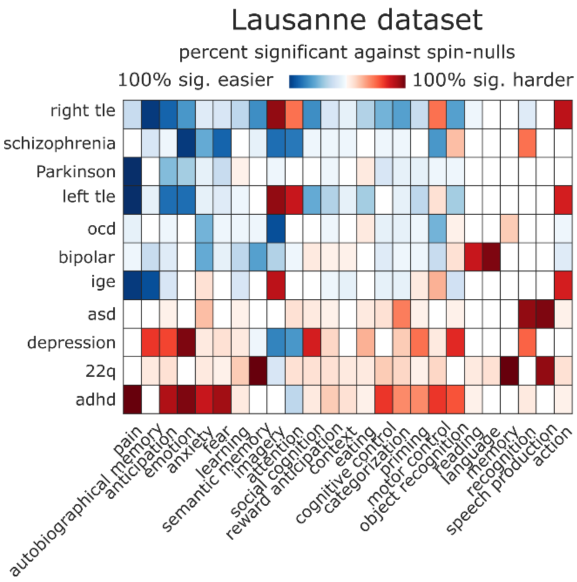


**Supplementary Fig. 10 | Effects of cortical thickness changes associated with each diagnostic category, for alternative connectome dataset and alternative cortical parcellation.** Heatmap shows how each diagnostic category reshapes the transition energy required to reach a given cognitive topography, presented as the percentage of transitions (out of all possible initial cognitive topographies) that are significantly facilitated (blue colour scale) or significantly dis-facilitated (red colour scale). Significance is assessed against a null distribution of randomly rotated cortical thickness alteration maps with preserved mean, variance, and spatial autocorrelation, such that the only differences with the original map are the neuroanatomical locations of increases and decreases, for the Lausanne DSI dataset. adhd = attention deficit/hyperactivity disorder; asd = autistic spectrum disorder; ocd = obsessive-compulsive disorder; ige = idiopathic generalised epilepsy; right tle = right temporal lobe epilepsy; left tle = left temporal lobe epilepsy.

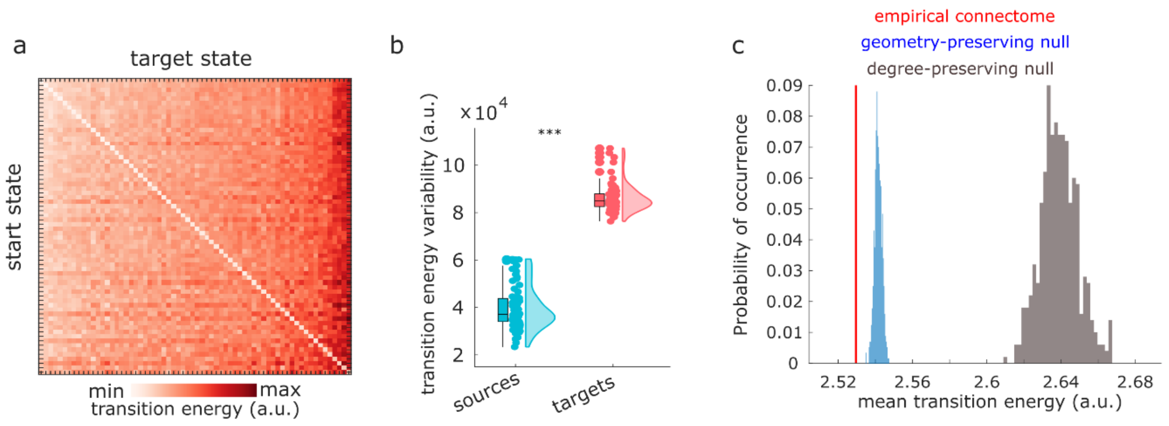


**Supplementary Fig. 11 I Replication with cognitive topographies defined by BrainMap. a** Transition energy between each pair of n = 66 cognitive topographies from BrainMap. **b** Variability (standard deviation) of transition energy is greater along target states than along start states (n=66). Box-plots: center line, median; box limits, upper and lower quartiles; whiskers, 1.5×interquartile range; ***, $p < 0.001$ from independent-samples t-tests (two-sided). **c** Degree-preserving randomised null models (grey) and null models that preserve the exact degree sequence and the approximate length distribution (blue) are significantly less favourable than the empirical human connectome (red) to support transitions between cognitive topographies defined by BrainMap.

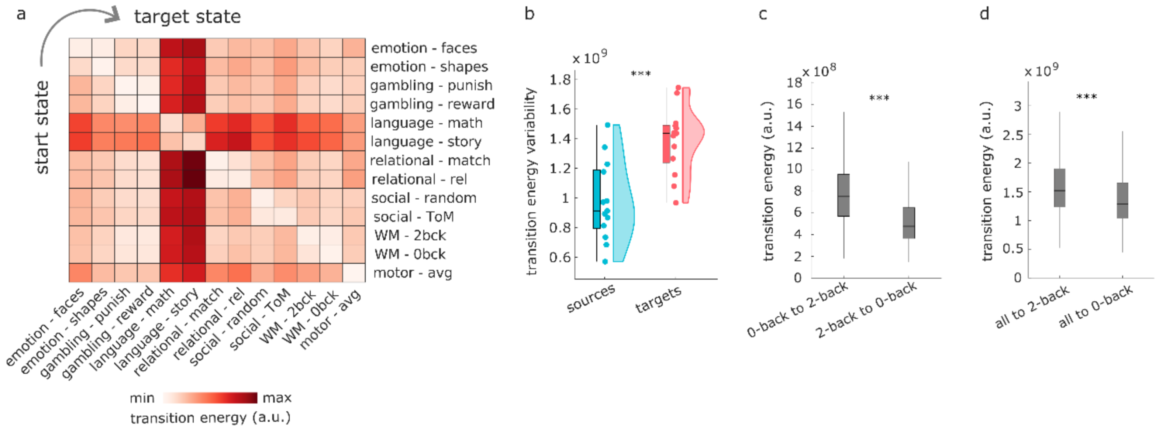


**Supplementary Fig. 12 | Transition energy between individual-level cognitive topographies defined from in-scanner task contrasts. a** Transition cost (energy) between each pair of 13 cognitive topographies defined as individual-specific task contrasts from 7 different tasks, across n=989 HCP participants. Rows indicate source states, columns indicate target states. **b** Variability (standard deviation) of transition energy is greater along the column dimension (target states, n=13) than along the row dimension (source states, n=13); $t(24) = 4.24$, $p < 0.001$, Cohen's $d = 1.61$. Box-plots: center line, median; box limits, upper and lower quartiles; whiskers, 1.5× interquartile range; ***, $p < 0.001$ from independent-samples t-tests (two-sided). **c** Transition cost (energy) is greater when transitioning from the subjectively easier 0-back working memory task to the subjectively more demanding 2-back working memory task, than the reverse ($t(985) = 20.58$, $p < 0.001$, Cohen's $d = 0.50$, n=986 participants). Box-plots: center line, median; box limits, upper and lower quartiles; whiskers, 1.5× interquartile range; ***, $p < 0.001$ from paired-samples t-tests (two-sided). **d** Transition cost (energy) is greater when transitioning from any task to the demanding 2-back working memory task, than from any task to the easier 0-back task ($t(951) = 13.16$, $p < 0.001$, Cohen's $d = 0.26$, n=952 participants). Box-plots: center line, median; box limits, upper and lower quartiles; whiskers, 1.5× interquartile range; ***, $p < 0.001$ from paired-samples t-tests (two-sided).

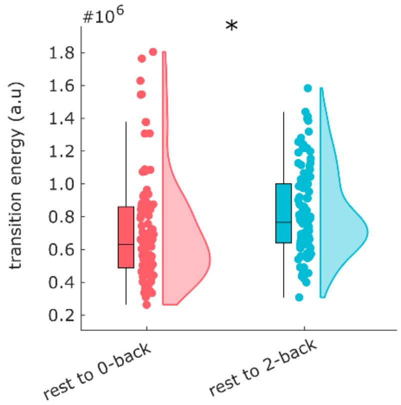


**Supplementary Fig. 13 | Energy for empirical transitions between temporally adjacent blocks during the working memory scan.** We computed the energy corresponding to each transition between temporally adjacent rest and task blocks, for each of the n=100 unrelated individuals in the HCP dataset. On average, transitions from rest blocks to subsequent 0-back blocks require less energy than transitions from rest blocks to subsequent 2-back blocks ($t(99) = 2.52$, $p = 0.013$, Cohen's $d = 0.39$). Box-plots: center line, median; box limits, upper and lower quartiles; whiskers, 1.5× interquartile range; ***, $p < 0.05$ from paired-samples t-test (two-sided).

## Supplementary tables

**Supplementary Table 1 | Participant-level comparison against null networks.** Statistical comparison (from paired-samples t-tests, two-sided) between participant-level overall transition energy distributions, for the empirical human connectome (n = 100 Human Connectome Project participants) and corresponding degree-preserving and degree- and cost-preserving rewired nulls.

| | Mean1 | SD1 | Mean2 | SD2 | df | t-score | p-value | Effect Size |
|---|---|---|---|---|---|---|---|---|
| empirical vs geometry-preserving | 1.25e+06 | 13400 | 1.26e+06 | 12200 | 99 | -21.49 | <0.001 | -0.77 |
| empirical vs degree-preserving | 1.25e+06 | 13400 | 1.35e+06 | 9160 | 99 | -60.18 | <0.001 | -9.05 |
| degree- vs geometry-preserving | 1.35e+06 | 9160 | 1.26e+06 | 12200 | 99 | 58.92 | <0.001 | 8.7 |

**Supplementary Table 2 | Participant-level results for alternative implementation of network control theory.** Statistical comparison (from paired-samples t-tests, two-sided) between participant-level overall transition energy distributions, for the empirical human connectome (n = 100 HCP participants) and corresponding degree-preserving and degree- and cost-preserving rewired nulls, for network control with time horizon T = 3 and network normalisation factor $c = 0.01 \times |\lambda(A)_{max}|$.

| | Mean1 | SD1 | Mean2 | SD2 | df | t-score | p-value | Effect Size |
|---|---|---|---|---|---|---|---|---|
| empirical vs geometry-preserving | 1.27e+06 | 9560 | 1.28e+06 | 9630 | 69 | -15.03 | <0.001 | -1.1 |
| empirical vs degree-preserving | 1.27e+06 | 9560 | 1.35e+06 | 11300 | 69 | -44.91 | <0.001 | -7.3 |
| degree- vs geometry-preserving | 1.35e+06 | 11300 | 1.28e+06 | 9630 | 69 | 36.34 | <0.001 | 6.3 |

**Supplementary Table 3 | Participant-level results for alternative implementation of network control theory.** Statistical comparison (from paired-samples t-tests, two-sided) between participant-level overall transition energy distributions, for the empirical human connectome (n = 100 HCP participants) and corresponding degree-preserving and degree- and geometry-preserving rewired nulls, for network control with NeuroSynth maps normalised to unit Euclidean norm.

| | Mean1 | SD1 | Mean2 | SD2 | df | t-score | p-value | Effect Size |
|---|---|---|---|---|---|---|---|---|
| empirical vs geometry-preserving | 1.3e+06 | 17200 | 1.31e+06 | 14900 | 99 | -17.38 | <0.001 | -0.76 |
| empirical vs degree-preserving | 1.3e+06 | 17200 | 1.42e+06 | 8480 | 99 | -64.11 | <0.001 | -8.6 |
| degree- vs geometry-preserving | 1.42e+06 | 8480 | 1.31e+06 | 14900 | 99 | 65.11 | <0.001 | 8.7 |

**Supplementary Table 4 | Participant-level results for the Lausanne dataset.** Statistical comparison (from paired-samples t-tests, two-sided) between participant-level overall transition energy distributions, for the empirical human connectome (n = 70 participants from the Lausanne dataset) and corresponding degree-preserving and degree- and cost-preserving rewired nulls.

| | Mean1 | SD1 | Mean2 | SD2 | df | t-score | p-value | Effect Size |
|---|---|---|---|---|---|---|---|---|
| empirical vs geometry-preserving | 3.03e+05 | 7640 | 2.99e+05 | 7460 | 99 | 14.81 | <0.001 | 0.49 |
| empirical vs degree-preserving | 3.03e+05 | 7640 | 2.65e+05 | 4140 | 99 | 50.37 | <0.001 | 6.1 |
| degree- vs geometry-preserving | 2.65e+05 | 4140 | 2.99e+05 | 7460 | 99 | -47.26 | <0.001 | -5.6 |

**Supplementary Table 5 | Participant-level results for Schaefer-parcellated data. Statistical** comparison (from paired-samples t-tests, two-sided) between participant-level overall transition energy distributions, for the empirical human connectome (n = 100 participants from the Human Connectome Project) and corresponding degree-preserving and degree- and cost-preserving rewired nulls.

| | Mean1 | SD1 | Mean2 | SD2 | df | t-score | p-value | Effect Size |
|---|---|---|---|---|---|---|---|---|
| empirical vs geometry-preserving | 35500 | 312 | 35800 | 298 | 99 | -27.19 | <0.001 | -0.84 |
| empirical vs degree-preserving | 35500 | 312 | 38100 | 260 | 99 | -61.91 | <0.001 | -9.02 |
| degree- vs geometry-preserving | 38100 | 260 | 35800 | 298 | 99 | 58.64 | <0.001 | 8.3 |

**Supplementary Table 6 | NeuroSynth terms.** Terms that overlapped between the NeuroSynth database [8] and the Cognitive Atlas [62] were included in the analysis.

| | | | | |
|---|---|---|---|---|
| action | adaptation | addiction | anticipation | anxiety |
| arousal | association | attention | autobiographical memory | balance |
| belief | categorization | cognitive control | communication | competition |
| concept | consciousness | consolidation | context | coordination |
| decision | decision making | detection | discrimination | distraction |
| eating | efficiency | effort | emotion | emotion regulation |
| empathy | encoding | episodic memory | expectancy | expertise |
| extinction | face recognition | facial expression | familiarity | fear |
| fixation | focus | gaze | goal | hyperactivity |
| imagery | impulsivity | induction | inference | inhibition |
| insight | integration | intelligence | intention | interference |
| judgment | knowledge | language | language comprehension | learning |
| listening | localization | loss | maintenance | manipulation |
| meaning | memory | memory retrieval | mental imagery | monitoring |
| mood | morphology | motor control | movement | multisensory |
| naming | navigation | object recognition | pain | perception |
| planning | priming | psychosis | reading | reasoning |
| recall | recognition | rehearsal | reinforcement learning | response inhibition |
| response selection | retention | retrieval | reward anticipation | rhythm |
| risk | rule | salience | search | selective attention |
| semantic memory | sentence comprehension | skill | sleep | social cognition |
| spatial attention | speech perception | speech production | strategy | strength |
| stress | sustained attention | task difficulty | thought | uncertainty |
| updating | utility | valence | verbal fluency | visual attention |
| visual perception | word recognition | working memory | | |

**Supplementary Table 7 | Nested terms.** Nested terms are more specific versions of the superordinate ones. Additionally, overlapping but not nested terms are also present, such as “sentence comprehension” and “language comprehension”, “speech perception” and “speech production”, or “face recognition” and “facial expression”; “cognitive control” and “motor control”.

| **Superordinate term** | **Nested terms** |
|---|---|
| Anticipation | reward anticipation |
| Attention | selective attention<br>spatial attention<br>sustained attention<br>visual attention |
| Decision | decision making |
| Emotion | emotion regulation |
| Imagery | mental imagery |
| Language | language comprehension |
| Learning | reinforcement learning |
| Memory | working memory<br>autobiographical memory<br>episodic memory<br>memory retrieval<br>semantic memory |
| Recognition | facial recognition<br>object recognition<br>word recognition |
| Response | response inhibition<br>response selection |
| Perception | visual perception |

**Supplementary Table 8 | BrainMap terms.** BrainMap terms are organized by behavioural domain. All 66 unique behavioural domain (excluding any undefined domains) used in analyses are shown here.

| | | | | |
|---|---|---|---|---|
| air-hunger | disgust | language | phonology | speech (action) |
| alcohol | emotion | learning | preparation | speech (language) |
| amphetamines | estrogen | marijuana | psychiatric medications | SSRIs |
| anger | execution | memory | reasoning | steroids and hormones |
| antidepressants | explicit | motion | rest | syntax |
| antipsychotics | fear | music | sadness | thermoregulation |
| anxiety | gustation | nicotine | semantics | thirst |
| attention | happiness | non-steroidal anti-inflammatory drugs | sexuality | time |
| audition | humour | observation | shape | vision |
| bladder | hunger | olfaction | sleep | working memory |
| caffeine | imagination | opioids | social cognition | |
| capsaicin | inhibition | orthography | soma | |
| cognition | interoception | pain | somesthesis | |
| colour | ketamine | pharmacology | space | |
