# Supplementary material for: Contributions of network structure, chemoarchitecture and diagnostic categories to transitions between cognitive topographies
Source: Nat Biomed Eng. 2024 Aug 5;8(9):1142–61. doi: 10.1038/s41551-024-01242-2 (PMC11410673; doi:10.1038/s41551-024-01242-2)
Supplement: Supplementary file 2 — Reporting Summary [file 41551_2024_1242_MOESM2_ESM.pdf]

nature portfolio

Corresponding author(s): Andrea Luppi

Last updated by author(s): Apr 14, 2024

Reporting Summary

Nature Portfolio wishes to improve the reproducibility of the work that we publish. This form provides structure for consistency and transparency in reporting. For further information on Nature Portfolio policies, see our Editorial Policies and the Editorial Policy Checklist.

Statistics

For all statistical analyses, confirm that the following items are present in the figure legend, table legend, main text, or Methods section.

n/a	Confirmed	
☐	☒	The exact sample size (*n*) for each experimental group/condition, given as a discrete number and unit of measurement
☐	☒	A statement on whether measurements were taken from distinct samples or whether the same sample was measured repeatedly
☐	☒	The statistical test(s) used AND whether they are one- or two-sided<br>*Only common tests should be described solely by name; describe more complex techniques in the Methods section.*
☐	☒	A description of all covariates tested
☐	☒	A description of any assumptions or corrections, such as tests of normality and adjustment for multiple comparisons
☐	☒	A full description of the statistical parameters including central tendency (e.g. means) or other basic estimates (e.g. regression coefficient) AND variation (e.g. standard deviation) or associated estimates of uncertainty (e.g. confidence intervals)
☐	☒	For null hypothesis testing, the test statistic (e.g. *F*, *t*, *r*) with confidence intervals, effect sizes, degrees of freedom and *P* value noted<br>*Give P values as exact values whenever suitable.*
☒	☐	For Bayesian analysis, information on the choice of priors and Markov chain Monte Carlo settings
☒	☐	For hierarchical and complex designs, identification of the appropriate level for tests and full reporting of outcomes
☐	☒	Estimates of effect sizes (e.g. Cohen's *d*, Pearson's *r*), indicating how they were calculated

Our web collection on statistics for biologists contains articles on many of the points above.

Software and code

Policy information about availability of computer code

Data collection	No new data were collected for this study, so no software was used for data collection.
Data analysis	Code for control energy computation in MATLAB (version 2019a was used) is available at https://github.com/gushiapi/Dynamic-Trajectory. DSI Studio is available at https://dsi-studio.labsolver.org. The Brain Connectivity Toolbox used for graph-theoretical properties and to generate degree-preserving null models is freely available at https://sites.google.com/site/betnet/. MATLAB code used to generate geometry-preserving null networks is freely available at https://www.brainnetworkslab.com/coderesources. The code for spin-based permutation testing of cortical correlations is freely available at https://github.com/frantisekvasa/rotate_parcellation. MRtrix3 is freely available at https://www.mrtrix.org/download/. The CONN toolbox (version 17f) is freely available at http://www.nitrc.org/projects/conn. Third-party Python software (version 3.8 was used) for Dominance Analysis is freely available at https://github.com/dominance-analysis/dominance-analysis. Third-party Python software for computing the network variance is available at https://github.com/rlambiot/variance. The ENIGMA toolbox (v1.1.3) is freely available at https://github.com/MICA-MNI/ENIGMA; Neuromaps (version 0.0.1) is freely available at https://netneurolab.github.io/neuromaps. We have made code available online at https://github.com/netneurolab/luppi-neurosynth-control.git.

For manuscripts utilizing custom algorithms or software that are central to the research but not yet described in published literature, software must be made available to editors and reviewers. We strongly encourage code deposition in a community repository (e.g. GitHub). See the Nature Portfolio guidelines for submitting code & software for further information.

Data

Policy information about availability of data

All manuscripts must include a data availability statement. This statement should provide the following information, where applicable:

- Accession codes, unique identifiers, or web links for publicly available datasets
- A description of any restrictions on data availability
- For clinical datasets or third party data, please ensure that the statement adheres to our policy

NeuroSynth meta-analytic maps are freely available from the NeuroSynth database at https://github.com/neurosynth/neurosynth. Human Connectome Project Young Adult resting-state, task-based and diffusion MRI data are available from https://www.humanconnectome.org/study/hcp-young-adult. Diffusion MRI data for the Human Connectome Project in DSI Studio-compatible format are available at http://brain.labsolver.org/diffusion-mri-templates/hcp-842-hcp-1021. The Lausanne structural connectivity dataset is available at https://doi.org/10.5281/zenodo.2872623. The ENIGMA cortical thickness data are provided as part of the ENIGMA Toolbox (v1.1.3), available at https://github.com/MICA-MNI/ENIGMA. PET receptor and transporter maps are available at https://github.com/netneurolab/hansen_receptors. Healthy cortical thickness and cerebral blood flow maps are available from Neuromaps at https://netneurolab.github.io/neuromaps. Source data for the figures are provided with this paper.

Research involving human participants, their data, or biological material

Policy information about studies with human participants or human data. See also policy information about sex, gender (identity/presentation), and sexual orientation and race, ethnicity and racism.

Reporting on sex and gender	Gender data were not collected separately from biological sex data. Analyses included invididuals of both sexes, as sex-related differences were not among the research hypotheses.
Reporting on race, ethnicity, or other socially relevant groupings	No groupings by race, ethnicity or socieconomic status were performed. For the HCP dataset, recruiting efforts were used by the HCP consortium to ensure that participants broadly reflect the ethnic and racial composition of the United States population as represented in the 2,000 decennial census.
Population characteristics	HCP data: 100 healthy participants (54 females and 46 males), mean age = 29.1 + 3.7 years. Lausanne data: 70 healthy participants (25 females, 45 males), age 28.8 + 8.9 years old.
Recruitment	No new data were collected for this study. See Van Essen et al., 2012, for recruitment of HCP participants.
Ethics oversight	The WU-Minn HCP Consortium (consortium of US and European intitutions led by Washington University and the University of Minnesota) approved the study protocol.<br>Lausanne dataset: Informed consent was obtained from all participants and the protocol was approved by the EthicsCommittee of Clinical Research of the Faculty of Biology and Medicine, University of Lausanne.

Note that full information on the approval of the study protocol must also be provided in the manuscript.

Field-specific reporting

Please select the one below that is the best fit for your research. If you are not sure, read the appropriate sections before making your selection.

☒ Life sciences ☐ Behavioural & social sciences ☐ Ecological, evolutionary & environmental sciences

For a reference copy of the document with all sections, see nature.com/documents/nr-reporting-summary-flat.pdf

Life sciences study design

All studies must disclose on these points even when the disclosure is negative.

Sample size	Sample sizes of individual datasets were not chosen as only open-source data were used. ENIGMA datasets were chosen as the maximum number of disorders with open data (as far as the authors were aware at the time of the analyses).
Data exclusions	No exclusions among the HCP 100 unrelated participants. Also no participants from the Lausanne dataset were excluded. Only Neurosynth terms in the intersection with the Cognitive Atlas were retained, since Neurosynth terms include a very wide variety of terms including regions ("dorsolateral") and clinical terms {"ADHD"), many of which are not relevant to the research question of defining cognitive topographies.
Replication	The analyses were repeated using a different structural connectivity dataset {Lausanne}, for which the replication was successful. The results were also successfully replicated using the BrainMap dataset, for a second probability matrix of terms (used to replace Neurosynth). Replication was also carried out with the broader set of N = 989 HCP participants.
Randomization	No randomization was performed, as the study did not include experimental groups.
Blinding	No blinding was performed, as the study did not include experimental groups.

Reporting for specific materials, systems and methods

We require information from authors about some types of materials, experimental systems and methods used in many studies. Here, indicate whether each material, system or method listed is relevant to your study. If you are not sure if a list item applies to your research, read the appropriate section before selecting a response.

Materials & experimental systems

n/a	Involved in the study
☒	☐ Antibodies
☒	☐ Eukaryotic cell lines
☒	☐ Palaeontology and archaeology
☒	☐ Animals and other organisms
☒	☐ Clinical data
☒	☐ Dual use research of concern
☒	☐ Plants

Methods

n/a	Involved in the study
☒	☐ ChIP-seq
☒	☐ Flow cytometry
☐	☒ MRI-based neuroimaging

Magnetic resonance imaging

Experimental design

Design type

Diffusion-weighted MRI, resting-state fMRI, and task-based fMRI.

Design specifications

HCP dataset: The diffusion MRI scan was conducted on a Siemens 3T Skyra scanner using a 2D spin-echo single-shotmultiband EPI sequence with a multi-band factor of 3 and monopolar gradient pulse. The spatial resolution was1.25 mm isotropic. TR=5500 ms, TE=89.50ms. The b-values were 1000, 2000, and 3000 s/mm2. The totalnumber of diffusion sampling directions was 90, 90, and S0 for each of the shells in addition to 6 bO images.
Functional data: gradient-echo EPI, TR= 720 ms, TE= 33.1 ms, flip angle = 52°, FOV= 208 × 180, voxel size = 2 mm isotropic. Resting-state data were collected, as well as task-based data pertaining to 7 tasks. Details can be found in Van Essen et al (2013).

Lausanne dataset: The protocol included (1) a magnetization-prepared rapid acquisition gradient echo {MPRAGE)sequence sensitive to white/gray matter contrast {1 mm in-plane resolution, 1.2 mm slice thickness), and (2) a diffusionspectrum imaging {DSI} sequence (128 diffusion-weighted volumes and a single b0 volume, maximum b-value 8 000 s/mm2, 2.2 x 2.2 x 3.0 mm voxel size).

Behavioral performance measures

HCP dataset: behavioural measures were collected by the HCP consortium, but not used in this study. For the in-scanner task data, we did not look at task performance.

Lausanne dataset:no behavioural measures were recorded during scanning.

Acquisition

Imaging type(s)

Diffusion and functional (resting-state, task-based)

Field strength

3T

Sequence & imaging parameters

HCP dataset: The diffusion MRI scan was conducted on a Siemens 3T Skyra scanner using a 2D spin-echo single-shotmultiband EPI sequence with a multi-band factor of 3 and monopolar gradient pulse. The spatial resolution was1.25 mm isotropic. TR=5500 ms, TE=89.50ms. The b-values were 1000, 2000, and 3000 s/mm2. The totalnumber of diffusion sampling directions was 90, 90, and 90 for each of the shells in addition to 6 b0 images.

Functional data: gradient-echo EPI, TR= 720 ms, TE= 33.1 ms, flip angle = 52°, FOV= 208 × 180, voxel size = 2 mm isotropic.

Lausanne dataset: The protocol included (1) a magnetization-prepared rapid acquisition gradient echo {MPRAGE) sequence sensitive to white/gray matter contrast {1 mm in-plane resolution, 1.2 mm slice thickness), and (2) a diffusionspectrum imaging {DSI} sequence {128 diffusion-weighted volumes and a single b0 volume, maximum b-value 8,000 s/mm2, 2.2 x 2.2 x 3.0 mm voxel size).

Area of acquisition

Whole brain (both datasets).

Diffusion MRI ☒ Used ☐ Not used

Parameters

HCP: The b-values were 1,000, 2,000, and 3,000 s/mm2. The total number of diffusion sampling directions was 90, 90 and 90 for each of the shells in addition to 6 bO images. 1.25 mm isotropic resolution.Lausanne: 128 diffusion-weighted volumes and a single bO volume, maximum b-value 8,000 s/mm2, 2.2 x 2.2 x 3.0 mm voxel size.

Preprocessing

Preprocessing software	HCP diffusion data for the 100 unrelated participants were pre-processed using DSI Studio. HCP diffusion data for the 989-participants dataset were pre-processed using MRtrx3. HCP resting-state functional data were pre-processed with the CONN toolbox. Lausanne data were provided already preprocessed {as connectivity matrices) using Connectome Mapper and Freesurfer. See https://doi.org/10.5281/zenodo.2872623 for further details about data processing. HCP task-based functional data were minimally pre-processed as per [123] and fixed-effects analyses were conducted using FSL's fMRI Expert Analysis Tool (FEAT).
Normalization	Normalization to MNI-152 template using the nonlinear registration algorithm implemented in the statistical parametric mapping (SPM). See Glasser et al., 2013 (NeuroImage) for details on HCP minimal preprocessing pipelines.
Normalization template	Desikan-Killiany anatomical atlas. Replication was performed with the Schaefer-100 atlas.
Noise and artifact removal	The minimally pre-processed DWI HCP data [48] were corrected for eddy-current and susceptibility artifacts. For the task-based data, we used the minimally pre-processed HCP data. See original publication for details (Glasser et al., 2013). The anatomica CompCor (aCompCor) method was used for denoising rs-fMRI fMRI data.<br><br>The aCompCor method involves regressing out of the functional data the following confounding effects: the first five principal components attributable to each individual's white-matter signal, and the first five components attributable to individual cerebrospinal fluid (CSF) six subject-specific realignment parameters (three translations and three rotations) as well as their first-order temporal derivatives. Linear de-trending was also applied, and the subject-specific de-noised BOLD-signal time series were band-pass-filtered to eliminate both low-frequency drift effects and high-frequency noise, thus retaining frequencies between 0.008 and 0.09 Hz.
Volume censoring	No volume censoring was used in this study.

Statistical modeling & inference

Model type and settings	We used two types of network null models: degree-preserving, and degree-preserving and cost-preserving. For each null model, we generated a population of 500 null networks starting from the empirical connectome, and computed the control energy between each pair of cognitive brain states from NeuroSynth, as done for the empirical connectome. We compared the overall control energy between all possible states obtained from the empirical connectome and from the distribution of null instances. We used permutation-based non-parametric t-tests to compare the transition energy to versus from each cognitive topography, and to perform subject-level comparisons of total transition energy against null networks.
Effect(s) tested	Difference between transition energy to and from each cognitive topography. Difference between total transition energy obtained from empirical versus null networks. Structural and anatomical predictors of transition energy to a given map. Difference between transition energy to different task-defined cognitive topographies.

Specify type of analysis: ☒ Whole brain ☐ ROI-based ☐ Both

Statistic type for inference<br>(See Eklund et al. 2016)	No voxel-level or cluster-level analyses were performed.
Correction	No voxel-level or cluster-level analyses were performed, so no such correction was requried.

Models & analysis

n/a	Involved in the study
☐	☒ Functional and/or effective connectivity
☐	☒ Graph analysis
☐	☒ Multivariate modeling or predictive analysis

Functional and/or effective connectivity	Functional connectivity was obtained as the regularized inverse covariance between regional time series.
Graph analysis	Both participant-level and group-level analysis were performed. Weighted structural connectivity graphs were used. Participation coefficient, binary degree, weighted degree and network-based variance were computed.
Multivariate modeling and predictive analysis	We used these characterizations of the NeuroSynth maps (correlation with connectome graph-theoretic properties; correlation with the cortical hierarchy; and network variance) as predictors against the average energy required to transition to each cognitive brain state. We performed multiple partial correlations, using each characterization in turn as predictor (after partialling out the effects of mean and traditional variance of each NeuroSynth map). As an alternative approach, to consider all predictors together and evaluate their respective contributions, we performed a dominance analysis with all five predictors. Dominance analysis seeks to determine the relative contribution 'dominance' of each independent variable to the overall fit (adjusted R2)) of the multiple linear regression model [4]. This is done by fitting the same regression model on every combination of predictors {2*p-1 submodels for a model with p predictors). Total dominance is defined as the average of the relative increase in R2 when adding a single predictor of interest to a

submodel, across all 2*p — 1 submodels. The sum of the dominance of all input variables is equal to the total adjusted R2 of the complete model, making the percentage of relative importance an intuitive method that partitions the total effect size across predictors.
